# Structural and functional dissection of neutralisation differences among SARS-CoV-2 variants using antigenicity prediction and CR3022 binding analysis

**DOI:** 10.3389/fimmu.2026.1810369

**Published:** 2026-07-06

**Authors:** Jyoti Sawant, Ajit Patil, Madhuri Thakar, Swarali Kurle

**Affiliations:** ICMR-National Institute of Translational Virology and AIDS Research, Pune, India

**Keywords:** antigenicity, B-cell epitopes, CR3022, immune escape, neutralizing antibodies, RBD mutations, SARS-CoV-2, vaccine-induced immunity

## Abstract

**Background:**

Progressive waves of coronavirus disease 2019 (COVID-19) have been driven by severe acute respiratory syndrome coronavirus 2 (SARS-CoV-2) variants carrying mutations in the spike glycoprotein, particularly in immunodominant regions such as the receptor-binding domain (RBD) and the N-terminal domain (NTD). These mutations can alter antigenic surfaces and are associated with changes in antibody recognition and vaccine-induced protection. Integrating experimental neutralisation data with computational analyses may contextualise variant-associated differences in antibody responses.

**Methods:**

Neutralisation responses were evaluated using a pseudovirus-based luciferase reporter assay including SARS-CoV-2 spike proteins from B.1 (Wuhan), B.1.617.2, AY.2 (Delta), and B.1.1.529 (Omicron). Plasma samples from three cohorts: naturally infected (I), vaccinated (V), and vaccinated-infected individuals (V+I), along with RBD-directed monoclonal antibody CR3022, were assessed to determine neutralizing titres (NT50). In parallel, spike sequences were analysed using epitope prediction, antigenicity profiling, and structural modelling. Docking simulations of CR3022 with variant RBDs were performed using HADDOCK, and binding parameters were estimated using PRODIGY.

**Results:**

Neutralisation responses varied across cohorts and viral variants, reflecting differences in immune exposure history. Plasma from V group individuals showed comparatively higher neutralisation titre, whereas B.1.617.2 and B.1.1.529 exhibited reduced susceptibility to neutralisation by infection-elicited antibodies. Computational analyses indicated variant-associated differences in predicted antigenicity and epitope landscapes within the RBD and NTD. Structural modelling and docking suggested that spike mutations may influence the CR3022-RBD interaction interface, with corresponding changes in predicted binding affinity across variants. These computational observations provide structural context for experimentally observed trends in reduced neutralisation but do not establish a direct mechanistic relationship.

**Conclusion:**

This study provides a combined experimental and computational characterization of SARS-CoV-2 variant-specific neutralisation across infection, vaccination and hybrid immunity-driven cohorts. The integration of pseudovirus neutralisation data with structural and in silico analyses offers a hypothesis-generating framework to contextualize observed differences in antibody responses and epitope recognition across variants.

## Introduction

The continued evolution of Severe Acute Respiratory Syndrome Coronavirus 2 (SARS-CoV-2) has resulted in the emergence of multiple variants with increased transmissibility and varying degrees of immune evasion. Variants of SARS-CoV-2 harbour mutations primarily within the spike glycoprotein that can alter antigenicity and influence susceptibility to neutralizing antibodies (Nabs) generated following prior infection or vaccination (1–4). Over successive epidemic waves, variants of concern, including B.1.1.7 (Alpha), B.1.315 (Beta), B.1.617.2 (Delta), and B.1.1.529 (Omicron), accumulated lineage-specific mutations that modified the antigenic landscape of the spike protein and antibody recognition (5).

The receptor-binding protein (RBD) and N-terminal domain (NTD) of the spike protein represent the major targets of the Nabs; mutations within these regions have been associated with altered humoral immune recognition (6–8). Changes in spike antigenicity resulting from amino acid substitution can influence antibody binding and neutralisation sensitivity (9–11). For example, the N439K mutation has been associated with increased binding affinity for human angiotensin-converting enzyme 2 (hACE-2) and reduced susceptibility to certain neutralizing antibodies (12). In contrast, antibodies directed against relatively conserved regions within the RBD, NTD or S2 subunit may retain partial cross-variant activity, highlighting the heterogeneity of epitope conservation and functional antibody recognition across SARS-CoV-2 variants (13). Studies evaluating combined RBD and NTD targeting antibodies have further suggested that multi-epitope targeting may help restrict viral escape (14).

Neutralisation assay using a pseudotyped viral system has emerged as a reliable and safe technique for evaluating variant-specific antibody responses. Polyclonal plasma neutralisation reflects the combined effects of prior antigen exposure, vaccination history, and affinity maturation and diverse antibody specificities, whereas monoclonal antibodies (mAbs) provide an opportunity to examine epitope-specific interactions and potential differences in antibody recognition among viral variants (15, 16). Among these, CR3022 is an RBD-directed mAb reported to recognize a relatively conserved but cryptic epitope within the SARS-CoV-2 spike protein (17). Previous studies have reported variable neutralisation activity of CR3022 against SARS-CoV-2, including proposed mechanisms involving conformational effects and altered epitope accessibility, highlighting the complexity of CR3022-mediated interactions.

While differences in neutralisation susceptibility among ancestral SARS-CoV-2, B.1.617.2, and B.1.1.529 variants have been extensively characterized in previous studies, less is understood about how these functional differences manifest across distinct real-world immune exposure histories, particularly in populations shaped by sequential waves of infection, vaccination, and breakthrough infection.

India experienced multiple pandemic waves dominated by distinct SARS-CoV-2 variants, providing an opportunity to examine immune responses generated in defined clinical cohorts representing natural infection, vaccination and hybrid immunity in different epidemiological settings. In particular, the widespread use of the ancestral spike-based ChAdOx1 nCoV-19/Covishield vaccine during successive variant waves offers a relevant framework for evaluating variant-specific neutralisation patterns in exposed cohorts. While reduced neutralisation sensitivity of emerging SARS-CoV-2 variants has been widely documented, comparatively fewer studies have integrated cohort-specific neutralisation profiling with structural analyses aimed at exploring potential mechanisms.

In the present study, we combined pseudovirus-based neutralisation assays with in silico epitope prediction, structural modelling, and exploratory docking analyses to investigate the relationship between spike mutations and antibody recognition. Plasma samples from infected, vaccinated, and vaccinated-infected (hybrid immunity) individuals were tested against pseudoviruses bearing spike proteins from B.1 (Wuhan), B.1.617.2, AY.2 (Delta), and B.1.1.529 (Omicron) variants. In addition, the RBD-specific monoclonal antibody CR3022 was evaluated using both *in vitro* neutralisation and computational interaction analyses to explore potential variant-dependent differences in binding behaviour. We further integrated structural interpretation of antibody-spike interactions with emphasis on the CR3022-associated conserved epitope region to provide a preliminary mechanistic context for observed neutralisation patterns. These analyses are intended to be hypothesis-generating and not confirmatory.

Overall, the computational analyses were used to complement the experimentally derived neutralisation data by providing structural context for observed variant-associated differences in antibody recognition across the studied cohorts.

## Materials and methods

**Figure 1:**
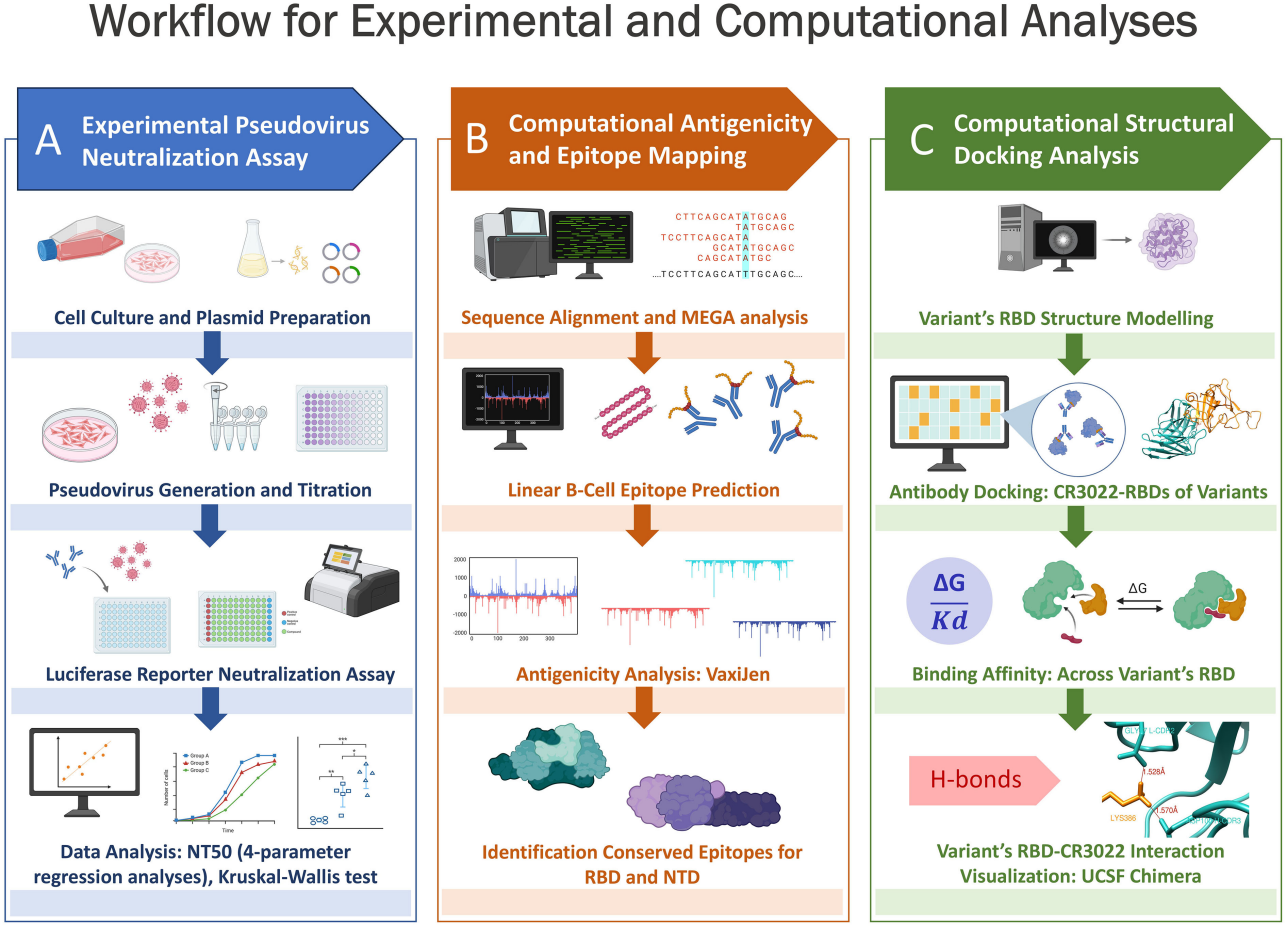
Integrated experimental and computational workflow of the study. Section **(A)** experimental workflow illustrating pseudovirus generation using spike constructs corresponding to SARS-CoV-2 variants, followed by pseudovirus quantification and neutralisation assays performed using plasma samples from infected (I), vaccinated (V), and vaccinated-infected (V+I) cohorts, along with the RBD-specific monoclonal antibody CR3022. Section **(B)** computational sequence-based analysis workflow used to predict antigenicity, linear B-cell epitopes, and conserved immunoreactive regions across SARS-CoV-2 variants, aimed at supporting comparative interpretation of experimental findings. Section **(C)** structural modelling and docking workflow depicting homology modelling of variant RBD structures, CR3022-RBD docking simulations, interface interaction analysis, and estimation of binding parameters. These analyses were performed as exploratory approaches to provide structural context for observed differences in antibody recognition across variants.

An integrated experimental and computational approach was used to characterize neutralisation of SARS-CoV-2 variants. A schematic overview of the methodological workflow is presented in **Figure 1**. Together, these modules provide an integrated framework that links experimental neutralisation outcomes with computational predictions of antigenicity, epitope stability, and structural determinants of immune escape.

### Section A (experimental)

#### Cell lines and culture conditions

HEK 293T (ATCC CRL 3216) and HEK-293 T-hACE2 Cell Line (BEI Resources, NIAID, NIH: NR-52511) were cultured in Dulbecco’s modified Eagle’s medium (DMEM) supplemented with 10% foetal bovine serum (FBS) and 0.5% penicillin-streptomycin. The ACE-2 receptor expression on the HEK-293 T-hACE2 cells was confirmed by flow cytometry using FITC-conjugated Anti-ACE2 Antibody (Sino Biological, 10108-MM37-F).

#### Plasmids and cloning

The lentivirus packaging plasmids pHAGE-CMV-Luc2-IRES-ZsGreen-W (NR-52516), HDM-Hgpm2 (NR-52517), HDM-tat1b (NR-52518) and pRC-CMV-Rev1b (NR-52519) and SARS-CoV-2 spike protein-expressing plasmids like B.1 (NR-53742), B.1.1.529 (NR-56470), B.1.1.7 (NR-55304), and B.1.351 (NR-55305) were obtained from BEI Resources, NIAID, NIH. The B.1.617.2 (Delta AY.2) spike plasmid (VG40819-UT) was obtained from Sino Biologicals. Plasmids were transformed into competent *E. Coli* (New England Biolabs, C2987H) and expanded in Luria Broth with antibiotic selection.

#### Human plasma specimens and monoclonal antibodies

The study was approved by the ICMR-National AIDS Research Institute Ethics Committee (NARI EC/2021-21). Written informed consent was obtained from all participants. The study included three groups defined according to immune exposure history.

1. Infected (I group): SARS-CoV-2-infected individuals from the first COVID-19 wave in India (May-September 2020), N = 25 (median age 42 years, M/F =14/11).
2. Vaccinated (V group): Individuals with no known prior infection, sampled after the second dose of Covishield (ChAdOx1-nCOV or AZD1222) (March to August 2021), N = 25 (median age 38 years, M/F =13/12).
3. Vaccinated + Infected (V+I group): Individuals infected during the third wave after receiving two doses of Covishield (ChAdOx1-nCOV or AZD1222) (April to July 2022), N = 25 (median age 37 years, M/F =14/11).

Detailed demographic characteristics, immune exposure history, enrolment chronology, and epidemiological context of the study cohorts are provided in **Supplementary Table 1**. The plasma samples were heat-inactivated at 56 °C for 30 minutes. Anti-SARS-CoV-2 spike IgG antibodies were confirmed using the Spike Trimer IgG ELISA kit (Acro Biosystems RAS-T048).

The monoclonal antibody CR3022 (NR-52392) targets the RBD, isolated from a SARS-CoV convalescent donor (BEI Resources, NIAID, NIH). Additional neutralisation analyses of variants were performed using monoclonal antibodies MW05 (Creative Diagnostics, DMABB-JX754) and NR-55295 (BEI Resources, NIAID, NIH).

#### Biosafety and ethical compliance

All experiments were performed under biosafety level 2 (BSL-2), in compliance with institutional policies. Human samples were anonymized to ensure confidentiality before processing, and all procedures complied with the Declaration of Helsinki and institutional guidelines.

#### Generation and titration of the SARS-CoV-2 variant pseudoviruses

Pseudoviruses expressing SARS-CoV-2 spike variants were generated using the lentiviral system incorporating the Luc2-IRES-ZsGreen reporter. HEK293T cells were seeded at a density of 6×10^5^cells/well in six-well plates. After 16–24 h, cells were co-transfected with

- 2 µg pHAGE-CMV-Luc2-IRES-ZsGreen-W (NR-52516)
- 0.22 µg Lentivirus packing plasmids (NR-52517, NR-52519, NR-52518)
- 0.68 µg of spike-expression plasmids

Transfection was performed using Calcium Phosphate ProFection^®^ System (Promega, E1200). At 16–18 h post-transfection, the media were replaced, and supernatants were harvested at 48 h, clarified by 0.45 µm filtration, aliquoted, and stored at -80 °C.

#### Pseudovirus titration

HEK-293T-hACE2 cells (2×10^4^ cells/well) were seeded in a 96-well plate and infected with neat or serial two-fold dilutions of the pseudovirus in the presence of Polybrene (5 µg/ml) (Sigma Aldrich, TR-1003). After 48 h, luciferase activity was quantified using substrate (Revvity, 6066761) Multimode Plate Reader (Victor X2, Perkin Elmer) at 540 to 640 nm. The pseudovirus titres were expressed as relative luminescence units per ml (RLU/ml).

#### Neutralisation assay

The neutralisation assays were performed using pseudoviruses bearing the B.1, B.1.617.2 and B.1.1.529 spike proteins for plasma and five variants (B.1, B.1.1.7, B.1.351, B.1.617.2 sublineage AY.2, and B.1.1.529) for monoclonal antibody CR3022. Heat-inactivated plasma samples were initially tested using serial dilutions ranging from neat to 1:64 in the pseudovirus neutralisation assay.

CR3022 antibody was evaluated across serial concentrations ranging from 10 µg/mL to 0.001 µg/mL. Plasma or CR3022 (25 µl) was incubated with pseudoviruses (25 µl of 4×10^6^ RLUs^/^mL) at 37 °C for 1 h. Subsequently, 50 µl HEK-293T-hACE2 cells (1×10^6^ cells/ml) were added, and plates were incubated for 48 h. Luminescence was measured as described above.

Neutralisation assays were performed using HEK-293 T-hACE2 Cell Line (up to passage 5). Consistent ACE2 expression was verified by flow cytometry using an anti-ACE2 monoclonal antibody, and validated cells were used for all experiments.

Samples exhibiting undetectable or poorly resolved NT_50_ values within this range were subjected to additional serial dilutions up to a reciprocal dilution of approximately 1:8192 (log_10_ 3.9134) to facilitate improved characterization of neutralizing activity and accurate NT_50_ estimation.

To assess reproducibility, 18% (n=14) of plasma samples representing a range of neutralisation responses were independently retested in duplicate and showed comparable NT_50_ values (**Supplementary Figure 3**). All neutralisation experiments with monoclonal antibodies (CR3022, MW05 and NR-55295) were independently performed twice with technical duplicates, and data are presented as mean ± standard deviation (SD).

#### Statistics

All experiments were performed with a sample size of at least two biological replicates. The average RLUs for each dilution were normalized to the cell and virus controls and used to calculate the neutralisation percentage.


$$(\%)\;\text{Neutralisation}=\left[1–\frac{\text{Normalized}\;\text{RLU}\;\text{of}\;\text{sample}}{\text{Normalized}\;\text{RLU}\;\text{of}\;\text{positive}\;\text{control}}\right]\times 100$$

Neutralisation titres (half-maximal inhibitory dilution; NT50) were determined using a log(inhibitor) versus normalized response variable-slope nonlinear regression model in GraphPad Prism version 8, with the top and bottom constraints fixed at 100 and 0, respectively. Percentage neutralisation values were plotted against log_10_ reciprocal plasma dilutions for curve fitting and NT_50_ prediction. Samples with poorly resolved NT_50_ values within the initial dilution range were further analysed by extending serial dilutions. Statistical comparisons between study groups were performed on log_10_-transformed NT_50_ values using the non-parametric Kruskal–Wallis one-way ANOVA test in GraphPad Prism v8.

### Section B (computational- antigenicity and epitope mapping)

#### B-cell epitopes prediction

The spike protein sequences of variants (B.1, B.1.1.7, B.1.351, B.1.617.2 sublineage AY.2, and B.1.1.529) were aligned using MEGA (Molecular Evolutionary Genetics Analysis). RBD domain, NTD, Stem Helix Residue, and Fusion Peptide regions were located and exported in FASTA format. Linear B-cell epitope predicted using four methods: ABCpred, BepiPred 2.0, Kolaskar & Tongaonkar and Ellipro Antibody Epitope prediction with a threshold of 0.5 (18–21). The change in antigenicity for SARS-CoV-2 Variants was determined for predicted linear B-cell epitopes. The epitopes predicted by the four methods with the antigenic properties were considered for further analysis.

#### Antigenicity estimation

The antigenicity of the full spike, NTD, RBD, Stem Helix Residue, Fusion Peptide, and predicted epitopes was assessed using VaxiJen 2.0 (threshold of 0.4). VaxiJen uses a physicochemical property-based auto and cross-covariance (ACC) translation of protein sequences and a partial least squares (PLS) algorithm for predicting protective antigen (22).

### Section C (computational- structural docking)

#### Homology modelling of the RBD structures of variants

The RBD amino acid sequences corresponding to the plasmids used in pseudovirus generation were identified within the plasmid sequence (Documents, BEI Resources, NIAID, NIH). Homology models were generated using SWISS-MODEL (template PDB:6W41) (23). Model selection criteria included sequence similarity, global model quality estimate (GMQE) value, qualitative model energy analysis (QMEAN) score, and a consensus-based distance constraint score (QMEANDisCo) (24).

#### Molecular docking of CR3022 with variant RBDs

We used the biomolecular modelling platform HADDOCK version 2.4, which conducts structure-based docking simulations (25, 26). The PDB tool was used to renumber the residues of the CR3022 antibody (PBD:6W41), ensuring that no residue IDs overlapped (27, 28). Active and passive residues were defined based on known interface residues from the literature (17, 29). The best docking clusters were selected based on HADDOCK scores and the RMSD of the lowest energy structure.

The docking analyses were performed using modelled isolated RBD structures and therefore represent simplified static interaction models that do not fully account for conformational flexibility or spike trimer dynamics.

#### Analysis of binding affinity and interactions between the RBDs and CR3022

The binding affinity (ΔG) and dissociation constant (Kd) for the docked complexes were predicted using Protein Binding Energy Prediction (PRODIGY) (30, 31). Hydrogen bonds and interface geometry were visualized UCSF Chimera (32).

## Results

### Section A (experimental)

#### Neutralisation of SARS-CoV-2 variant pseudoviruses by plasma samples

**Figure 2:**
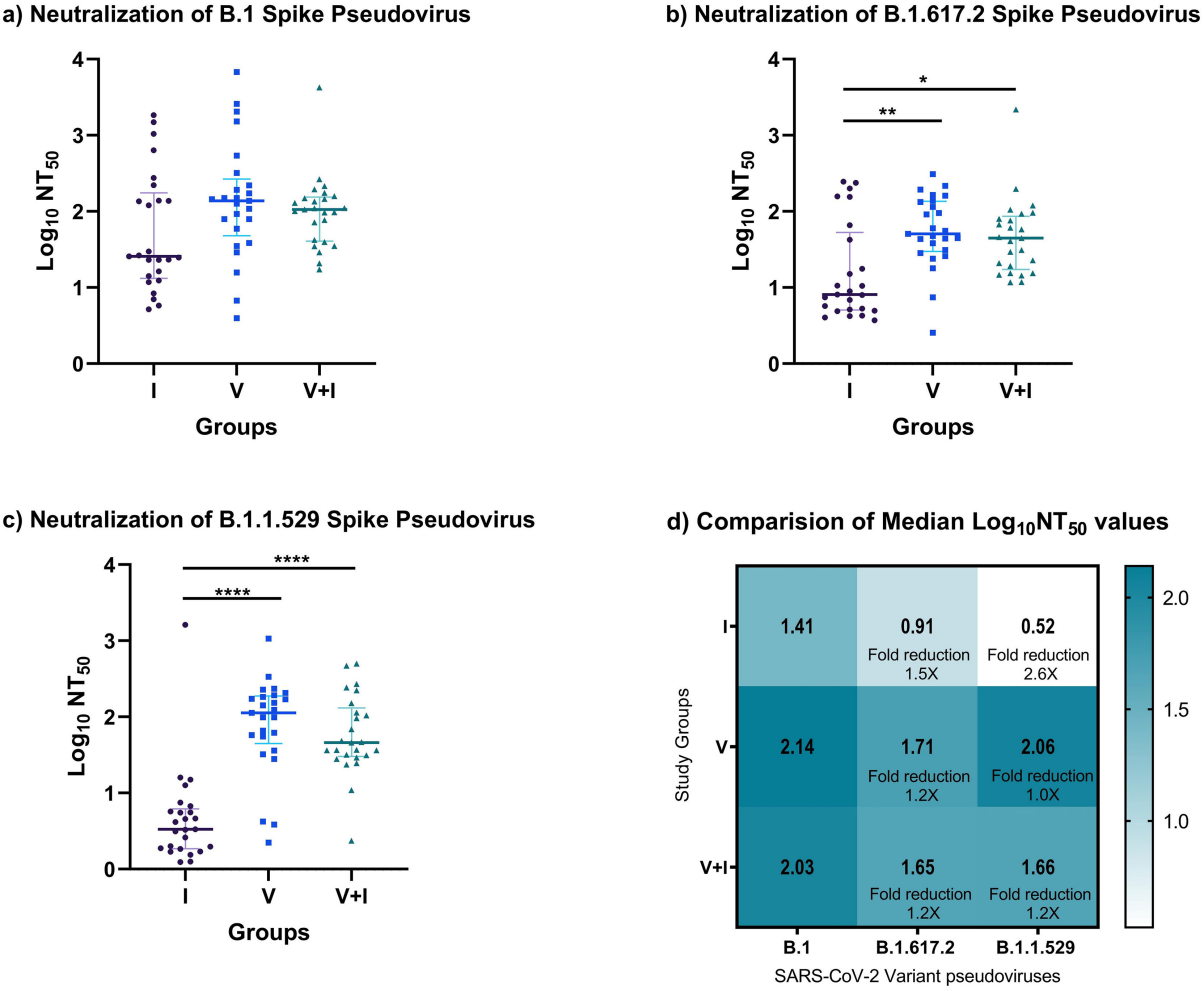
Neutralisation responses of plasma samples against SARS-CoV-2 variants B.1, B.1.617.2, and B.1.1.529. The Y-axis represents the Log_10_NT_50_ values, corresponding to the reciprocal plasma dilution resulting in 50% reduction of pseudovirus infection, and the X-axis represents three study groups: infected (I), vaccinated (V), and vaccinated-infected (V+I). Each point represents an individual sample, and horizontal lines indicate the median Log_10_NT_50_ and the interquartile range of the group. Statistical analysis was performed using the Kruskal-Wallis non-parametric test. P values are represented as ^∗^p< 0.05, ^∗∗^p< 0.01, ^∗∗∗∗^p< 0.0001. **(a)** Neutralisation responses against B.1 spike pseudovirus: Neutralisation activity was detected across all groups. Although differences in median NT_50_ values were observed, no statistically significant differences were identified among the groups. **(b)** Neutralisation responses against B.1.617.2 spike pseudovirus: Neutralisation responses varied across groups, with lower median neutralisation observed in the I group relative to the V and V+I groups. These findings are presented as comparative trends and should not be interpreted as direct measures of immune escape. **(c)** Neutralisation responses against B.1.1.529 spike pseudovirus: Reduced neutralisation responses were observed in the I group relative to the V and V+I groups, with statistically significant differences where indicated. These findings reflect differences in pseudovirus susceptibility to plasma-mediated neutralisation under the experimental conditions used. **(d)** Heat Map of the median NT_50_ values of three groups: The colour intensity corresponds to the median NT_50_ magnitude (Darker colour: higher neutralizing activity, Lighter colour: lower neutralizing activity). The fold reduction in neutralisation compared to the B.1 is also demonstrated in the heat map among the three groups.

Plasma samples containing anti-spike IgG antibodies from three groups: I group, V group, and V+I group, were evaluated to neutralize pseudoviruses bearing spike proteins of three variants: B.1, B.1.617.2 sublineage AY.2, and B.1.1.529. The Neutralisation profiles of the study groups by variants are provided in **Supplementary Figure 1**. Plasma samples with undetectable or poorly resolved NT_50_ values within the initial assay dilution range were further analysed using extended serial dilutions, and the representative neutralisation curves are presented in **Supplementary Figure 2**. The neutralisation curves generated from the dilution range were subsequently subjected to nonlinear regression analysis for NT_50_ prediction and curve fitting accuracy. Neutralisation titres were expressed as Log_10_ NT_50_ values (**Figure 2**).

For the B.1 pseudovirus, all three groups demonstrated detectable neutralisation responses (**Figure 2a**). The median Log_10_NT_50_ values were 1.41 for the I group, 2.14 for the V group, and 2.03 for the V+I group (**Figure 2d**). Although the V and V+I groups exhibited relatively higher neutralisation titres compared with the I group, no statistically significant difference was observed among the groups.

Against the B.1.617.2 pseudovirus, a reduction in neutralisation titres was observed in comparison with the B.1 variant (Figure b). The median Log_10_NT_50_ values decreased to 0.91 in the I group, 1.71 in the V group, and 1.65 in the V+I group (Figure d). Statistical analysis revealed significantly higher neutralisation titres in the V group and in the V+I group compared with the I group (*p* = 0.0018) compared with the I group. The fold reduction in median neutralisation titres against B.1.617.2 relative to B.1 was approximately 1.5-fold in the I group and 1.2-fold in both the V and V+I groups.

The greatest reduction in neutralisation was observed against the B.1.1.529 pseudovirus (**Figure 2c**). The I group exhibited markedly lower neutralisation activity, with a median Log_10_NT_50_ value of 0.52, whereas the V and V+I groups showed median values of 2.06 and 1.66, respectively (**Figure 2d**). Neutralisation titres in both the V and V+I groups were significantly higher than those in the I group (*p* < 0.0001). Compared with the B.1 variant, the I group demonstrated an approximately 2.6-fold reduction in neutralisation against B.1.1.529, while the V and V+I groups showed relatively smaller reductions of approximately 1.0-fold and 1.2-fold, respectively.

Overall, the findings indicate that vaccination-induced and hybrid immunity-mediated NAbs retained stronger neutralizing activity against SARS-CoV-2 variants compared with infection-induced immunity alone. However, a progressive decline in neutralisation efficacy was observed from the B.1 to B.1.617.2 and B.1.1.529 variants, with B.1.1.529 showing the highest level of immune escape. These findings suggest progressive reduction in neutralisation sensitivity associated with spike protein alterations and differences in antibody response breadth across the study groups.

### Neutralisation of SARS-CoV-2 variants by monoclonal antibody CR3022

**Figure 3:**
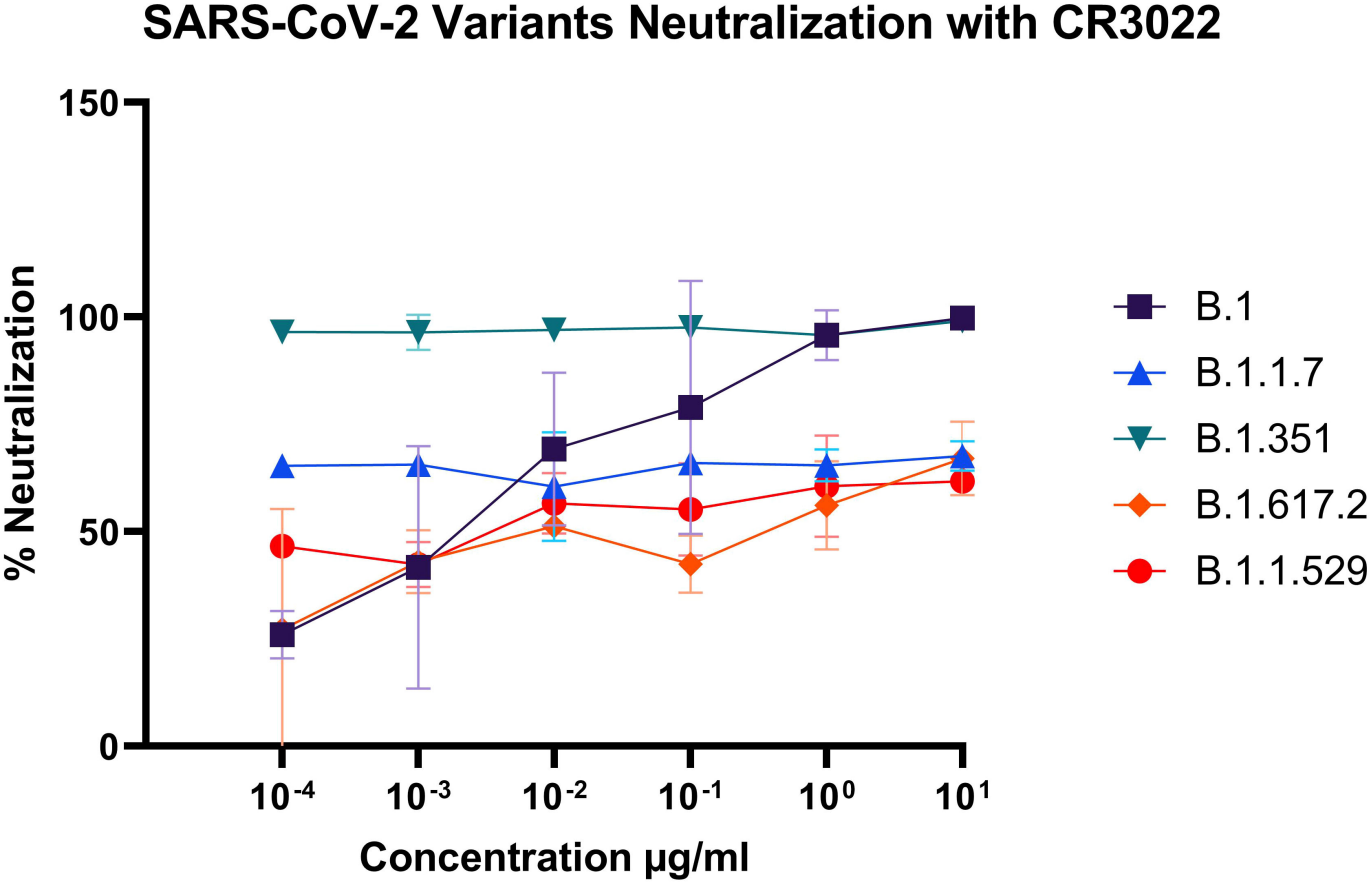
Neutralisation efficiency of RBD-binding monoclonal antibody CR3022 (NR-52392) against SARS-CoV-2 variants. The graph displays the percentage of neutralisation on the Y-axis for the SARS-CoV-2 variant pseudoviruses (B.1, B.1.1.7, B.1.351, B.1.617.2, and B.1.1.529) at increasing monoclonal antibody CR3022 concentrations (µg/mL) on the X-axis. B.1 and B.1.351 pseudoviruses were completely neutralized at a concentration of 10 µg/mL. In contrast, B.1.1.7, B.1.617.2, and B.1.1.529 pseudoviruses exhibited reduced neutralisation with a plateauing response even at higher antibody concentrations. Data represent mean ± SD from two independent experiments performed in duplicate.

To assess variant-specific effects on antibody recognition independent of polyclonal plasma antibody responses, RBD binding mAb, CR3022, was evaluated against the pseudoviruses bearing spike protein for B.1, B.1.1.7, B.1.351, B.1.617.2, and B.1.1.529 variants (**Figure 3**).

CR3022 demonstrated comparatively greater neutralisation against B.1 and B.1.351 pseudoviruses under the tested experimental conditions. In contrast, B.1.1.7, B.1.617.2, and B.1.1.529 showed reduced sensitivity, with maximal neutralisation ranging between 50 to 70% and incomplete neutralisation even at higher concentrations. These findings suggest variant-dependent differences in susceptibility to CR3022-mediated neutralisation, potentially reflecting altered epitope accessibility or conformational differences affecting antibody recognition.

To provide additional experimental context, comparative neutralisation analyses using monoclonal antibodies MW05 and NR-55295 against B.1, B.1.617.2, and B.1.1.529 pseudoviruses were also performed and are presented in the **Supplementary Figure 4**. These antibodies similarly demonstrated variant-dependent differences in neutralisation profiles, supporting the broader observation that spike mutations can differentially influence monoclonal antibody recognition across SARS-CoV-2 variants.

### Section B (computational- antigenicity and epitope mapping)

Considering the observed differences in neutralisation across SARS-CoV-2 variants using both polyclonal plasma and the monoclonal antibody CR3022, exploratory in silico analyses were performed to examine potential structural and antigenic features associated with these observations. Antigenicity prediction and B-cell epitope mapping were applied to identify both conserved and variant-associated predicted epitopes within the RBD and NTD that may provide context for differences in antibody recognition across variants. Structural visualization of representative conserved predicted epitopes was additionally performed to assess their approximate spatial distribution relative to previously reported antigenic regions. Because CR3022 has a defined RBD-binding specificity, molecular docking analyses were restricted to the RBD and were used to compare predicted antibody-RBD interaction patterns, including binding affinity estimates and hydrogen bonding interactions across variants. These computational analyses were intended to provide supportive and hypothesis-generating context for the experimental neutralisation findings rather than establish mechanistic determinants of antibody escape.

#### Antigenicity of spike protein and its regions for SARS-CoV-2 variants

**Figure 4:**
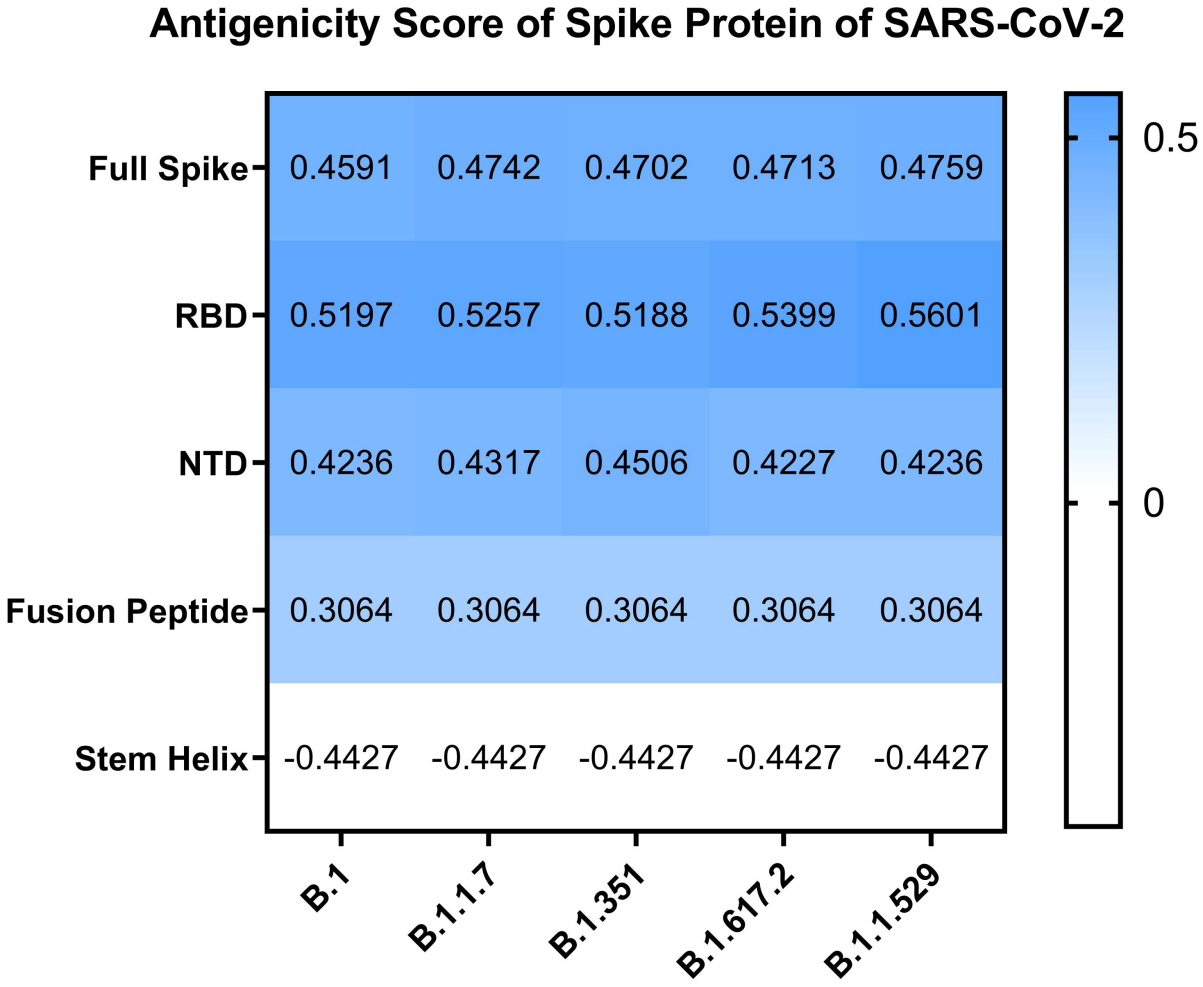
Predicted antigenicity of the SARS-CoV-2 spike protein and its structural regions across variants. Heatmap showing computationally predicted antigenicity scores for the full-length spike protein and selected spike regions, including the Receptor Binding Domain (RBD), N-terminal Domain (NTD), Fusion Peptide, and Stem Helix, of the SARS-CoV-2 variants. Antigenicity scores were calculated using the VaxiJen server, where higher positive values indicate higher predicted antigenicity and are represented by darker colour intensity. Regions with scores below the established antigenicity threshold are designated as non-antigenic and are shown in lighter colours. These scores represent sequence-based computational predictions and provide comparative estimates of antigenic potential; they should not be interpreted as direct measures of antibody binding, immunogenicity, or neutralisation susceptibility.

Predicted antigenicity analyses were performed using VaxiJen 2.0 with a threshold score of 0.4 for the full-length spike protein and selected spike regions, including the N-terminal domain (NTD), receptor-binding domain (RBD), Stem Helix, and Fusion Peptide (**Figure 4**).

Across the variants examined, the RBD exhibited higher predicted antigenicity scores than the full-length spike protein and NTD. In contrast, the Stem Helix and Fusion Peptide regions yielded scores below the selected antigenicity threshold. Modest variation in predicted antigenicity scores was observed among the SARS-CoV-2 variants for the spike protein and its constituent domains. Among the variants analysed, B.1.1.529 exhibited the highest predicted antigenicity scores for the full-length spike protein and RBD, whereas B.1.351 showed slightly higher predicted antigenicity within the NTD region. These results represent computational estimates of antigenic potential and do not directly reflect antibody binding or neutralisation activity.

#### Variant-dependent distribution of predicted B-cell epitopes across SARS-CoV-2 variants

Linear B-cell epitopes within the RBD and NTD were predicted using multiple computational approaches, including ABCpred, BepiPred 2.0, Kolaskar and Tongaonkar antigenicity, and ElliPro. Detailed epitope predictions and corresponding antigenicity scores are presented in **Supplementary Tables 2**, **3**, while representative conserved and variant-associated epitopes are summarized in **Tables 1**, **2**.

**Table 1:** Summary of representative conserved and variant-associated RBD epitopes predicted across SARS-CoV-2 variants.

Epitope region	Residues *	Variants detected	Putative RBD antibody class region**	Category	Observation
FKCYGVSPTKLNDLCF	377 - 392	B.1, B.1.351, B.1.617.2, B.1.1.529	Class 4 (CR3022-associated region)	Conserved	Predicted epitope overlapping the reported CR3022/Class 4 region
YGVSPTKLNDLCFTN(V)	380 - 394	B.1, B.1.351, B.1.617.2, B.1.1.529	Class 4 (CR3022-associated region)	Conserved	Predicted by ElliPro across multiple variants
PTKLNDLCFTNVYADS	384 - 399	B.1, B.1.1.7, B.1.351, B.1.617.2, B.1.1.529	Class 4 (CR3022-associated region)	Conserved	Predicted across all variants
EVRQIAPGQTGNIADY	406 - 421	B.1.351, B.1.617.2, B.1.1.529	Between Class 4 and Class 3 regions	Conserved	Predicted across later variants
TGCVIAWNSNNLDSKV	430 - 445	B.1, B.1.1.7, B.1.351, B.1.617.2, B.1.1.529	Class 3-associated surface	Conserved	Predicted across all variants
VVLSFELLHAPATVCG	511 - 526	B.1, B.1.1.7, B.1.351, B.1.617.2, B.1.1.529	Outside canonical Classes 1–4	Conserved	Predicted across all variants
RKSNLKPFERDISTEI	457 - 472	B.1, B.1.1.7, B.1.351, B.1.617.2, B.1.1.529	Adjacent to Class 4 region	Conserved	Predicted across all variants
YGVGYQPYRVVVLSFE	501 - 516	B.1.1.7, B.1.351	Class 1/2-associated surface	Conserved among selected variants	Predicted in B.1.1.7 and B.1.351
DSKVGGNYNYRYRLFR	442 - 457	B.1.617.2	Class 3-associated surface	Variant-associated	Predicted only in B.1.617.2
NYNYRYRLFRKSNLKP	448 - 463	B.1.617.2	Class 3-associated surface	Variant-associated	Predicted only in B.1.617.2
LRSYSFRPTYGVGHQP	492 - 507	B.1.1.529	Class 4-associated region	Variant-associated	Predicted only in B.1.1.529
SVLYNLAPFFTFKCYG	366 - 381	B.1.1.529	Class 4-associated region	Variant-associated	Predicted only in B.1.1.529

Conserved and variant-associated RBD epitopes identified by multiple prediction algorithms (ABCpred, BepiPred 2.0, Kolaskar & Tongaonkar, and ElliPro) are summarized to facilitate comparison of predicted antigenic regions among SARS-CoV-2 variants. Approximate residue positions are based on the Wuhan-Hu-1 reference spike sequence. Putative antibody class associations were assigned according to overlap with previously reported RBD antigenic regions, including the Class 4 CR3022-associated epitope, and are intended for contextual interpretation only. Detailed prediction outputs and corresponding antigenicity scores are presented in **Supplementary Table 2**. * Residue numbering corresponds to the Wuhan-Hu-1 spike sequence. ** Antibody class assignments are approximate and based on overlap with previously reported RBD antigenic regions. These assignments are intended for contextual interpretation and do not establish experimental antibody binding.

**Table 2:** Summary of representative conserved and variant-associated NTD epitopes predicted across SARS-CoV-2 variants.

Epitope region	Residues*	Variants detected	Putative NTD supersite region**	Category	Observation
CVNL(T/F)TRTQLPPAYT	15 - 28	B.1, B.1.1.7, B.1.351, B.1.617.2, B.1.1.529	N1 supersite	Conserved	Predicted epitope corresponding to the reported N1 supersite region
TTRTQLPPAYTNS(FTR)	19 - 31	B.1, B.1.1.7, B.1.351, B.1.617.2, B.1.1.529	N1 supersite	Conserved	Predicted across all variants with minor sequence variation
HVSGTNGTKRFD	69 - 80	B.1, B.1.1.7, B.1.351, B.1.617.2, B.1.1.529	Outside major supersite loops	Conserved	Conserved predicted NTD epitope identified by multiple methods
DLEGKQGNFKNLR	178 - 190	B.1, B.1.1.7, B.1.351, B.1.617.2, B.1.1.529	N3 supersite	Conserved	Predicted epitope corresponding to the reported N3 supersite region
RSYLTPGDSSSGWTA	246 - 260	B.1, B.1.351, B.1.617.2, B.1.1.529	N5 supersite	Conserved	Predicted epitope corresponding to the reported N5 supersite region
GTTLDSKTQ	107 - 115	B.1, B.1.1.7, B.1.351, B.1.617.2, B.1.1.529	N5 supersite	Conserved	Predicted epitope located within the reported N5 supersite region
VDLPIGINI/LPIGINI	227 - 235/229 - 235	B.1, B.1.1.7, B.1.351, B.1.617.2, B.1.1.529	Adjacent to N5 supersite	Conserved	Conserved predicted epitope adjacent to the N5 supersite region
EFVFKN blue	191 - 196	B.1.617.2	N3 supersite	Variant-associated	Predicted only in B.1.617.2 by the applied methods
KHTPIIVR red	206 - 214	B.1.1.529	Outside major supersite loops	Variant-associated	Predicted only in B.1.1.529 by the applied methods
PIIVREPEDLPQGFSA red	209 - 222	B.1.1.529	Outside major supersite loops	Variant-associated	Predicted only in B.1.1.529 by the applied methods

Conserved and variant-associated NTD epitopes identified by multiple prediction algorithms (ABCpred, BepiPred 2.0, Kolaskar & Tongaonkar, and ElliPro) are summarized to facilitate comparison of predicted antigenic regions among SARS-CoV-2 variants. Approximate residue positions are based on the Wuhan-Hu-1 reference spike sequence. Putative supersite assignments were made according to overlap with previously described NTD antigenic supersite regions (N1, N3, and N5) and are provided for contextual interpretation only. Detailed prediction outputs and corresponding antigenicity scores are presented in **Supplementary Table 3**. *Residue numbering corresponds to the Wuhan-Hu-1 reference spike sequence. **Supersite assignments are approximate and based on overlap with previously reported NTD antigenic supersite regions (N1, N3 and N5). These assignments are provided for contextual interpretation and do not establish experimental antibody binding.

Comparative analysis indicated differences in the distribution of predicted epitopes among the SARS-CoV-2 variants examined. Within the RBD, several predicted epitopes were detected across multiple variants, including regions encompassing EVRQIAPGQTGNIADY, TGCVIAWNSNNLDSKV, VVLSFELLHAPATVCG, RKSNLKPFERDISTEI, and PTKLNDLCFTNVYADS (**Table 1**). Several conserved predicted epitopes mapped to regions overlapping previously reported RBD antigenic surfaces (33), including regions associated with the reported CR3022/Class 4 epitope. Predicted epitopes encompassing FKCYGVSPTKLNDLCF, YGVSPTKLNDLCFTN, and PTKLNDLCFTNVYADS were identified within this region across multiple variants (**Table 1**).

Additional variant-associated predicted epitopes were identified in B.1.617.2 and B.1.1.529. These included DSKVGGNYNYRYRLFR and NYNYRYRLFRKSNLKP in B.1.617.2, as well as LRSYSFRPTYGVGHQP and SVLYNLAPFFTFKCYG in B.1.1.529. Several of these variant-associated epitopes were located within or adjacent to regions corresponding to previously described RBD antigenic surfaces (**Table 1**).

Similarly, analysis of the NTD identified several predicted epitopes that were consistently detected across multiple variants, including regions encompassing CVNL(T/F)TRTQLPPAYT, TTRTQLPPAYTNS(FTR), HVSGTNGTKRFD, DLEGKQGNFKNLR, RSYLTPGDSSSGWTA, and VDLPIGINI/LPIGINI (**Table 2**). Several of these conserved predicted epitopes mapped to regions corresponding to previously described NTD antigenic supersites (34), including N1, N3, and N5 supersite regions. Additional variant-associated predicted epitopes were identified in B.1.617.2 and B.1.1.529, including EFVFKN and HRSYLTPGDSSSGWT, respectively, while PIIVREPEDLPQGFSA was predicted only in B.1.1.529 (**Table 2**). Overall, the analyses identified both conserved and variant-associated predicted antigenic regions within the RBD and NTD across the SARS-CoV-2 variants examined.

**Figure 5:**
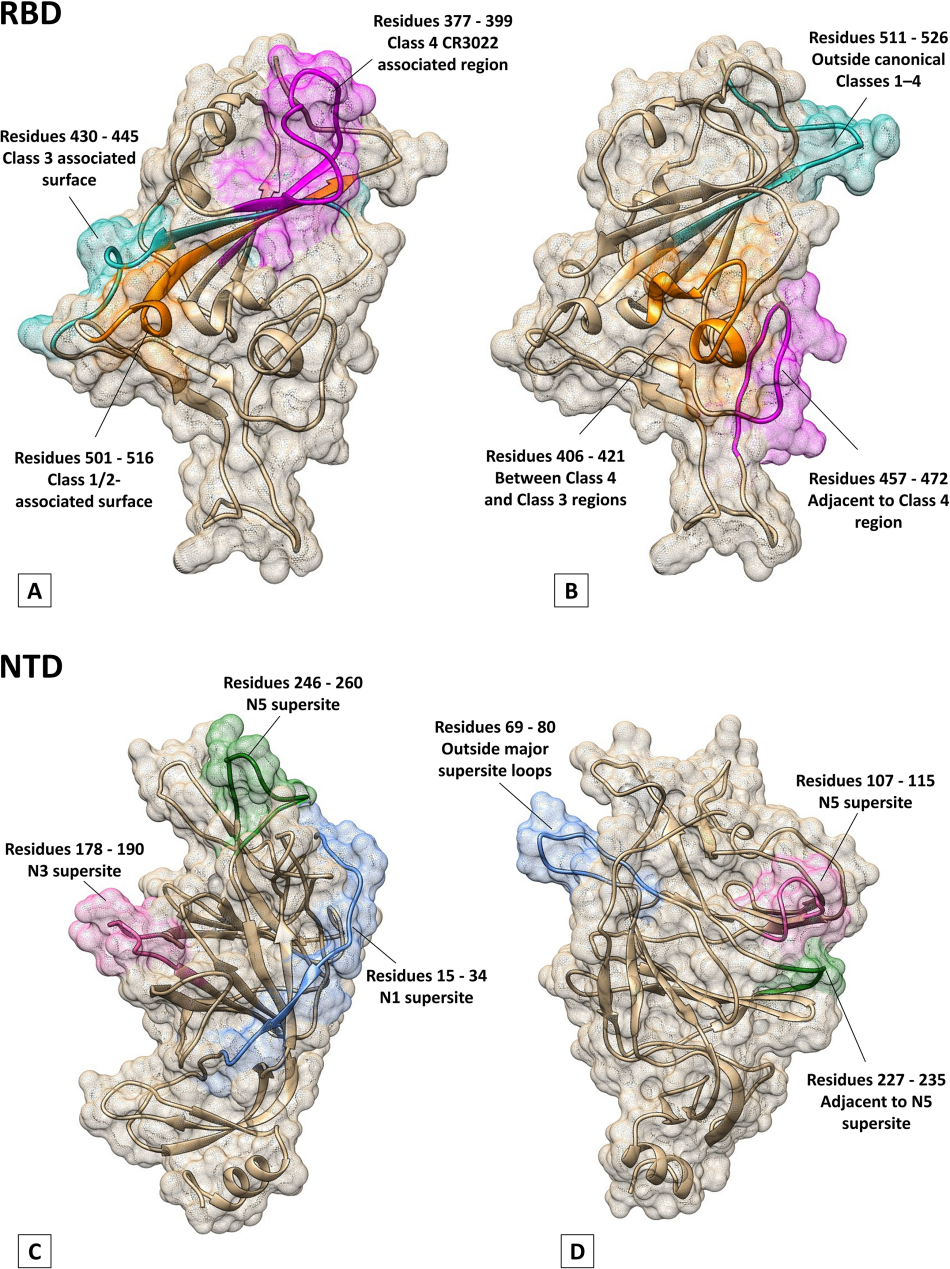
Structural visualization of representative conserved predicted B-cell epitope regions within the RBD and NTD of SARS-CoV-2 variants. Ribbon and surface representations of the spike RBD and NTD are shown, with representative conserved predicted B-cell epitope regions highlighted and annotated according to approximate residue positions. Structural regions are displayed to provide contextual comparison with previously described RBD antibody class-associated surfaces and NTD antigenic supersite regions. **(A, B)** Representative conserved predicted epitopes within the RBD identified across variants by multiple prediction methods. Highlighted regions include epitopes overlapping the reported Class 4 CR3022-associated region (residues 377-399), Class 3-associated surface (residues 430-445), regions positioned between Class 4 and Class 3-associated surfaces (residues 406-421), Class 1/2-associated surface (residues 501-516), and regions outside canonical Classes 1-4 (residues 511-526). **(C, D)** Representative conserved predicted epitopes within the NTD identified across variants by multiple prediction methods. Highlighted regions include predicted epitopes corresponding to previously described N1, N3, and N5 antigenic supersite regions, as well as adjacent or non-supersite regions. Residue numbering corresponds to the Wuhan-Hu-1 reference spike sequence (GenBank accession NC_045512). Structural visualization was performed using UCSF Chimera. Supersite and antibody class assignments are approximate and provided for contextual interpretation only; they do not establish experimental antibody binding or epitope classification.

Structural mapping of representative conserved predicted B-cell epitopes indicated that these regions were distributed across multiple surface-exposed regions of both the RBD and NTD (**Figure 5**). Within the RBD, conserved predicted epitopes were identified within residues 377-399, 406-421, 430-445, 457-472, 501-516, and 511-526. The epitope spanning residues 377–399 overlapped a region previously reported to include the conserved cryptic epitope recognized by class 4 antibodies, including CR3022. Predicted epitopes within residues 430–445 were mapped to Class 3-associated surface, and 457–472 mapped to regions adjacent to Class 4 region. Additional predicted epitopes were observed within residues 406–421 and 501-516, corresponding to regions positioned between reported antibody-associated surfaces class 1–4 regions.

Within the NTD, conserved predicted epitopes were distributed across residues 15-34, 69-80, 107-115, 178-190, 227-235, and 246-260. Several predicted regions overlapped or were adjacent to previously described NTD antigenic supersites, including N1, N3, and N5, whereas other predicted epitopes were located outside the major supersite loops.

Although several predicted epitopes occupied broadly similar structural regions across variants, sequence differences were observed between B.1.617.2 and B.1.1.529 within selected RBD and NTD epitope regions, indicating redistribution of the predicted epitope landscape rather than complete conservation of epitope composition. Representative variant-associated predicted epitopes identified in B.1.617.2 and B.1.1.529 were additionally visualized structurally to provide contextual comparison of their spatial distribution relative to reported antigenic regions (**Supplementary Figure 5**). These structural representations provide contextual support for the predicted epitope distribution.

### Section C (computational- structural docking)

#### modelling Structural and docking of CR3022 with variant RBDs

High-confidence structural models of variant RBDs were generated and validated (**Supplementary Table 4**). All RBD structural models demonstrated acceptable stereochemical quality and confidence scores. To further explore potential structural differences associated with CR3022 recognition, molecular docking was performed between the CR3022 and modelled RBD structures from B.1, B.1.1.7, B.1.351, B.1.617.2, and B.1.1.529 variants using HADDOCK 2.4 (**Supplementary Table 5**).

Docking analysis indicated variant-dependent differences in predicted CR3022-RBD interactions (**Table 3**). CR3022 binding to the B.1 RBD was used as the reference interaction, exhibiting a predicted binding affinity of -17.0 kcal/mol, and a dissociation constant (Kd) of 3.4e-13 M. Modest reductions in predicted binding affinity were observed for B.1.1.7 and B.1.617.2 (–16.4 kcal/mol). In contrast, B.1.351 showed a comparatively stronger predicted binding affinity (-17.7 kcal/mol) with a lower predicted Kd value, whereas B.1.1.529 exhibited a reduced predicted binding affinity (-14.9 kcal/mol) accompanied by a comparatively higher predicted Kd (1.2e-11 M).

**Table 3:** CR3022 antibody and RBD of the SARS-CoV-2 variants interaction metrics: Binding affinity (kcal/mol) represents estimated Gibbs free energy of binding derived from computational docking analyses.

Complexes	Binding affinity (kcal/mol)	Change in binding affinity (ΔΔG)	Dissociation constant (Kd) (M) at 25.0°C
B.1 RBD - CR3022	-17.0	–	3.4e-13
B.1.1.7 RBD - CR3022	-16.4	-0.6	9.1e-13
B.1.351 RBD - CR3022	-17.7	0.7	9.6e-14
B.1.617.2 RBD - CR3022	-16.4	-0.6	9.5e-13
B.1.1.529 RBD - CR3022	-14.9	-2.1	1.2e-11

The binding affinity change (ΔΔG) relative to B.1 RBD-CR3022 complex indicates differences in predicted binding affinity across variants; negative ΔΔG values suggest comparatively reduced predicted binding affinity with respect to the reference structure. The dissociation constant (Kd), where applicable, provides an additional estimate of binding interaction strength, with lower values indicating relatively stronger predicted binding affinity within the computational framework.

**Figure 6:**
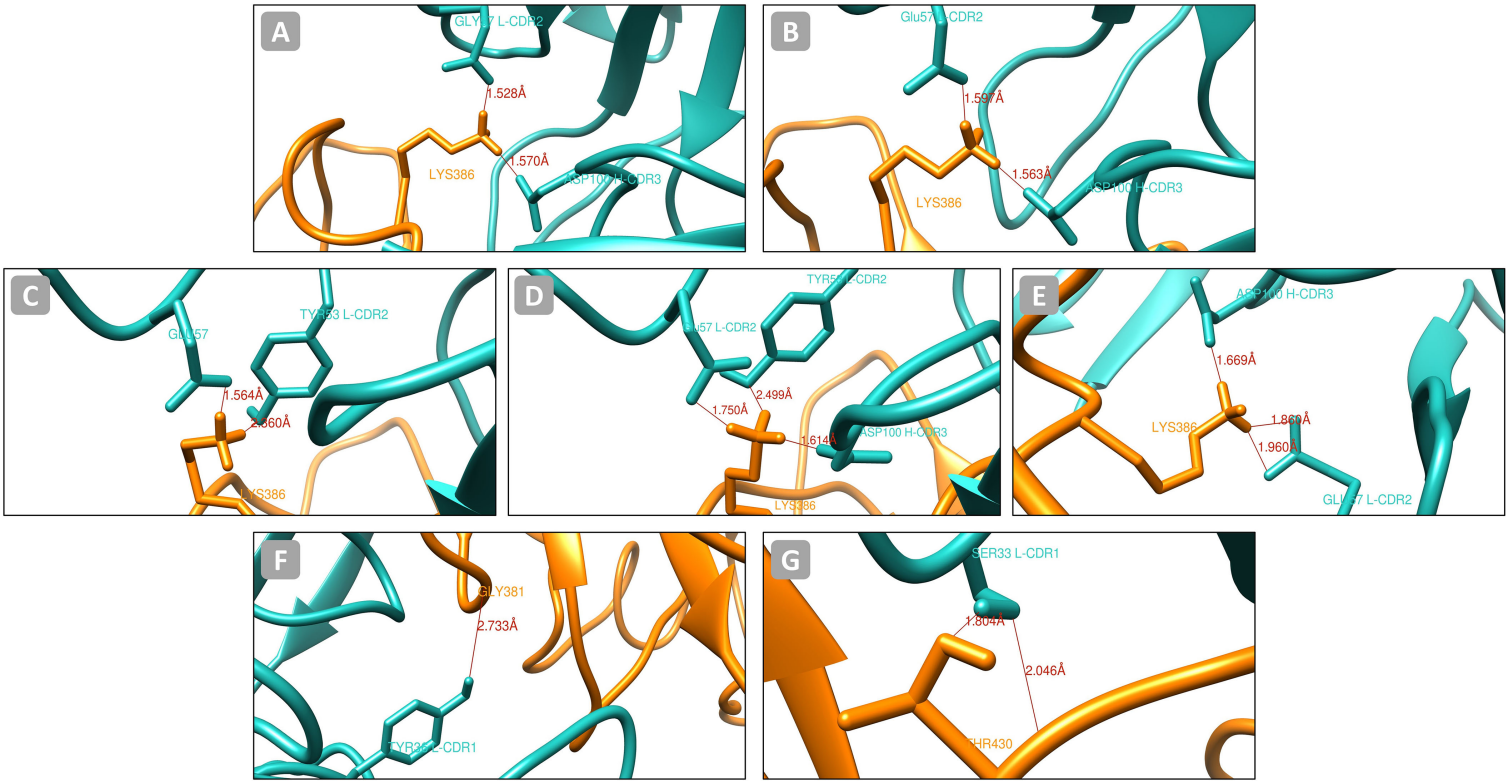
Predicted H-bond interactions between monoclonal antibody CR3022 (PDB ID: 6W41, teal green colour) and modelled RBD region (orange colour) of pseudoviruses of SARS-CoV-2 variants: The molecular docking analyses were performed using HADDOCK version 2.4, and predicted H-bond interactions within the resulting RBD-CR3022 complexes analysed using UCSF Chimera. The interactions involving the RBD residue Lys386 and the CR3022 residues were examined for comparative analysis among the variants. **(A, B)** In B.1 and B.1.351 RBD-CR3022 complexes, predicted H-bond interactions were observed between Lys386 of the RBD and Asp100 of H-CDR3, as well as Glu57 of L-CDR2, with comparatively shorter bond lengths relative to other variants. **(C)** In the B.1.1.7-RBD-CR3022 complex, Lys386 was predicted to interact with Tyr53 of L-CDR2 instead of Asp100 H-CDR3. **(D)** In the B.1.1.529 RBD-CR3022 complex, Lys386 was predicted to interact with Asp100, Glu57, and Tyr53 through three H-bonds. **(E)** In B.1.617.2 RBD-CR3022 complex, comparatively longer predicted H-bond were observed between Lys386 and Asp100 or Glu57 of CR3022. **(F)** Predicted variant-specific interactions between Gly381 of the B.1.1.7 RBD and Tyr36 of L-CDR1. **(G)** Predicted interactions between Thr430 of B.1.351 RBD and Ser33 of CR3022 through two H-bonds.

**Figure 7:**
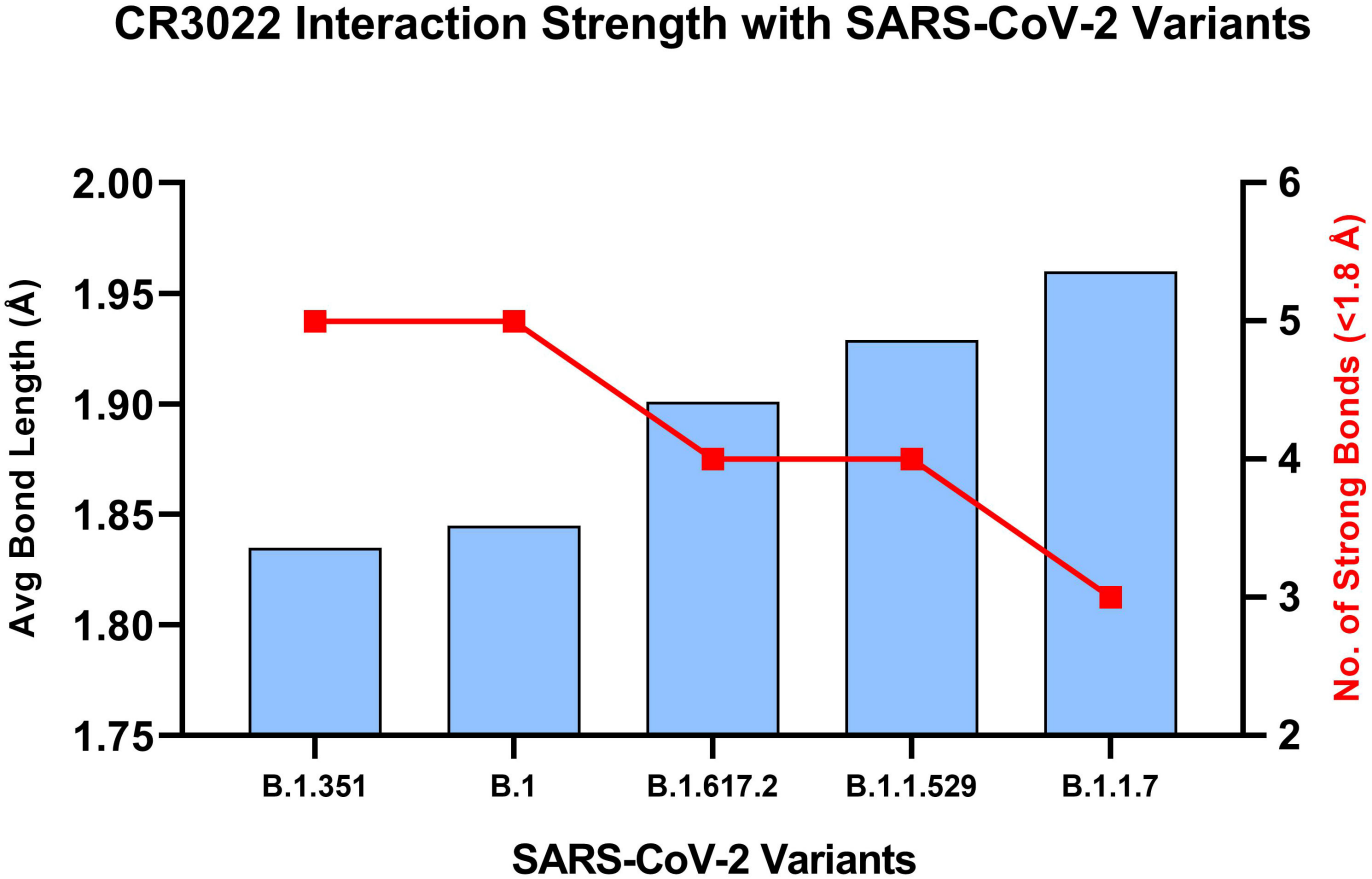
Predicted Interaction characteristics between monoclonal antibody CR3022 and RBD of SARS-CoV-2 variants: patterned bars (left Y-axis) represent the average predicted H-bond length (Å) calculated from identified H-bonds (1.5 Å and 2.5 Å) between CR3022 and the RBD of the various SARS-CoV-2 variants B.1.351, B.1, B.1.617.2, B.1.1.529, and B.1.1.7. The red line graph (right Y-axis) indicates the number of predicted strong H-bonds (≤ 1.8 Å) identified within each modelled complex. Comparatively higher average bond lengths and fewer strong predicted H-bonds were observed for the B.1.1.7 RBD-CR3022 complex, whereas the B.1.351 complex exhibited comparatively shorter average predicted bond lengths.

Analysis of intramolecular hydrogen bonds (H-bonds) using UCSF Chimera (32) identified variant-specific differences at the residues previously implicated in CR3022 recognition, including Lys386, Tyr380 and Thr430 (17, 35). Variations in the number of predicted H-bonds, interacting residues, and average bond lengths were observed across variants (**Figures 6**, **7**). In the B.1.1.7 RBD-CR3022 complex, predicted H-bonds involving Try380 of the RBD with Glu50 of H-CDR2, and Thr430 and Ser33 of L-CDR1 were not observed. In contrast, B.1.351 RBD-CR3022 complex exhibited two predicted H-bonds between Thr430 of RBD and Ser33 of CR3022, whereas a single interaction was observed in other variants. Variants B.1.617.2, B.1.1.529, and B.1.1.7, which demonstrated comparatively lower predicted binding affinities, also exhibited fewer strong predicted H-bonds and comparatively increased average bond lengths relative to B.1 and B.1.351.

These computational observations suggest potential differences in CR3022-RBD interaction patterns among variants. However, because the docking analyses were performed using isolated RBD structures, they provide only complementary structural information and do not fully account for the structural and dynamic features that influence antibody binding in the intact spike trimer.

## Discussion

This study integrated experimental pseudovirus neutralisation assays with complementary computational analyses to assess variant-associated differences in antibody recognition across SARS-CoV-2 variants. The present study does not aim to establish the novelty of reduced neutralisation sensitivity among SARS-CoV-2 variants, which has been extensively reported previously, but rather to investigate these responses within the studied cohorts and to explore their potential structural context using CR3022-centered analyses. Our findings suggest that differences in neutralisation may be associated with variant-specific alterations within the spike protein, particularly in the RBD and NTD, which represent major targets of neutralizing antibodies. These observations are consistent with the possibility that cumulative epitope-level changes contribute to differences in antibody recognition across variants.

Differences in immune exposure history may have contributed to the breadth of neutralisation responses observed across cohorts. Vaccinated and hybrid immunity groups showed comparatively broader neutralisation across variants than infection-derived responses. Covishield Vaccine (ChAdOx1 nCoV-19) delivers a controlled dose of prefusion-stabilized spike antigen, promoting consistent antigen presentation and avoiding viral immune evasion mechanisms. In contrast, natural infection exposes the host to the full spectrum of viral antigens, with varying antigen quantity depending on viral load, while simultaneously engaging immune-modulatory processes such as interferon suppression, altered antigen presentation and viral replication. These factors likely contribute to variable and less robust NAb and T-cell responses. Consistent with this, we observed broader cross-neutralisation in vaccinated individuals, whereas infected individuals showed limited activity against antigenically distant variants, including B.1.1.529 (**Figure 2c**), in agreement with prior reports (36, 37). However, interpretation of cohort differences should be made cautiously because the interval between last antigenic exposure and sample collection, prior undocumented infections, and differences in epidemiological background may contribute to variability in NT_50_ values.

Neutralisation responses against B.1.617.2 and B.1.1.529 pseudoviruses were lower than those observed against ancestral B.1 across study groups, with the largest reduction detected for B.1.1.529. Although reduced neutralisation of these variants has been reported previously, the present data provide cohort-specific experimental observations and place these findings within an exploratory structural framework. Importantly, no significant difference was observed between the V and V+I groups under the conditions tested, suggesting that vaccination-associated immunity contributed substantially to cross-variant neutralisation in this cohort.

The observed patterns of reduced neutralisation against SARS-CoV-2 variants are consistent with prior reports of immune escape across diverse populations (38–40), although differences in magnitude may reflect variation in exposure history, vaccination status, circulating variants, and assay methodologies (39, 41–44). These comparisons have been incorporated to emphasize that while immune escape trends are broadly conserved, quantitative neutralisation responses may vary across populations.

Neutralisation analyses using the monoclonal antibody CR3022 enabled assessment of variant-associated differences in antibody recognition independent of polyclonal plasma variability. Although the reported CR3022-binding region remained largely conserved across B.1, B.1.1.7, B.1.351, and B.1.617.2 (YNSASFSTFKCYGVSPTKLNDLCF), differential neutralisation profiles were observed (**Figure 3**), suggesting that conservation of epitope sequence alone may not necessarily translate into equivalent functional antibody recognition. Additional sequence changes within the corresponding region in B.1.1.529 (YNLAPFFTFKCYGVSPTKLNDLCF) coincided with further reduction in CR3022-mediated neutralisation. To provide additional experimental context, comparative neutralisation analyses using monoclonal antibodies MW05 and NR-55295 were included and similarly demonstrated variant-dependent neutralisation profiles (**Supplementary Figure 4**).

Previous studies have shown that CR3022 targets a conserved cryptic RBD epitope with limited accessibility in the prefusion spike trimer (45). Experimental evidence further suggests that CR3022 binds to the B.1.1.529 RBD, may remain partially preserved despite extensive mutations (46). However, reduced CR3022-mediated neutralisation observed in the present study suggests that preserved binding does not necessarily correspond to equivalent neutralisation activity and may additionally depend on epitope accessibility and spike structure. Structural cryo-EM studies of B.1.1.529 have shown that substitutions including S371L, S373P, and S375F can alter local RBD conformation and epitope accessibility (47–49). Accordingly, the reduced CR3022-mediated neutralisation observed in the present study may reflect the combined effects of structural changes and epitope sequence variation rather than direct loss of epitope identity alone.

To further explore these differences, computational docking analyses were performed to compare predicted antibody-RBD interaction patterns across variants (**Figures 6**, **7**). Differences in predicted hydrogen bonding and electrostatic interactions were observed at the antibody-RBD interface despite partial conservation of the CR3022 epitope sequence. In particular, mutations such as L452R and T478K present in B.1.617.2 have previously been associated with enhanced ACE2 receptor affinity and altered RBD surface properties, which could potentially influence antibody interaction geometry and Fab orientation (50–52). Similarly, in B.1.1.529, substitutions including S371L, S373P, S375F, Q493R, N440K, and G446S have been reported to remodel the RBD surface and alter local electrostatic or steric characteristics that may affect antibody recognition (53, 54). In addition, previous studies have also suggested that furin cleavage may generate heterogeneous spike conformations with altered epitope exposure, potentially contributing to reduced neutralisation sensitivity (55).

However, these findings should be interpreted cautiously. The docking analyses were performed using isolated RBD structures and provide a simplified representation of antibody-RBD interactions. Although comparatively stronger CR3022-RBD interactions were predicted, the docking-derived energies should not be interpreted as quantitative estimates of binding affinity or as direct evidence of biological activity. Experimentally determined binding affinities measured using surface plasmon resonance (SPR) and bio-layer interferometry (BLI) have been reported in the nanomolar range and may differ substantially from docking-derived estimates (46, 56). These discrepancies likely arise from the simplified and static nature of docking approaches and the inherent limitations of computational affinity prediction. In addition, the docking approach does not fully account for spike trimer architecture, conformational flexibility, steric accessibility, induced-fit effects, or dynamic stability of antibody-antigen complexes. Accordingly, the computational results are presented as supportive of possible interaction trends rather than mechanistic explanations for pseudovirus neutralisation or antibody escape.

Epitope prediction analyses identified a redistribution of predicted antigenic regions across variants together with retention of selected predicted epitopes. Comparison of the predicted RBD epitopes with previously described SARS-CoV-2 antigenic regions indicated that some conserved epitopes localized to regions associated with reported antibody classes. In particular, a conserved predicted epitope spanning residues overlapping the reported CR3022-associated region mapped to the previously described class 4 RBD surface (17, 33), whereas additional predicted epitopes localized to regions adjacent to previously described class 3 and receptor-binding motif associated antigenic surfaces (33, 57). Similarly, several conserved predicted NTD epitopes mapped to regions overlapping or adjacent to previously described NTD antigenic supersites, including N1, N3, and N5 regions, providing contextual support that selected predicted epitopes occupy previously recognized antigenic surfaces. Conserved epitopes that continued predicted antigenicity and surface accessibility across variants (**Figure 5**) may represent potential targets for cross-reactive antibody responses and warrant further experimental investigation. Structural visualization of representative variant-associated predicted epitopes further showed that B.1.617.2 and B.1.1.529 occupied broadly similar structural regions while exhibiting differences in amino acid composition, suggesting redistribution of predicted epitope composition rather than complete conservation of epitope identity (**Supplementary Figure 5**).

Interestingly, increased predicted antigenicity in emerging SARS-CoV-2 variants did not correspond to increased neutralisation susceptibility. As shown in **Figure 4**, B.1.617.2 and B.1.1.529 exhibited higher predicted antigenicity scores than B.1, and several predicted epitopes also demonstrated increased antigenicity scores (**Supplementary Tables 2**, **3**). However, both polyclonal plasma and CR3022-mediated neutralisation were reduced for these variants (**Figures 2**, **3**). These observations suggest that sequence-driven antigenicity alone may not predict functional antibody recognition and that factors such as conformational masking, glycan shielding, epitope accessibility, and structural organization may influence neutralisation outcomes (58, 59).

This study has several limitations. The sample size was limited, and not all circulating or emerging SARS-CoV-2 lineages were included in the experimental or computational analyses. We acknowledge that a key limitation of this study is the variability in the interval between the last antigenic exposure (infection or vaccination) and sample collection across cohorts, which may influence observed NT50 values due to ongoing antibody maturation and waning. Although sampling was conducted within relatively defined post-exposure windows, precise standardization across individuals was not feasible. In addition, the lack of complete information regarding prior asymptomatic SARS-CoV-2 infections may introduce unrecognized heterogeneity in baseline immunity. Computational analyses were exploratory and require validation using experimental structural and functional approaches. Epitope analyses focused primarily on the RBD and NTD and therefore do not capture the full antigenic landscape of the spike protein. Finally, neutralisation and computational findings were generated using *in vitro* and in silico approaches and do not directly predict *in vivo* protective immunity.

In conclusion, this study examined neutralisation responses in infection, vaccination, and hybrid immunity derived cohorts and explored their potential structural context through CR3022-centered analyses. While reduced neutralisation sensitivity of emerging SARS-CoV-2 variants has been widely documented, the present work provides cohort-specific experimental observations together with exploratory epitope mapping and structural analyses. The findings suggest that variant-associated differences in neutralisation may be influenced not only by epitope sequence variation but also by factors related to structural accessibility and conformational context. By integrating pseudovirus neutralisation data with epitope prediction and structural modelling, this study provides an exploratory framework for investigating variant-associated changes in antibody recognition and identifies conserved antigenic regions that merit further experimental evaluation.

## Data Availability

The original contributions presented in the study are included in the article/**Supplementary Material**. Further inquiries can be directed to the corresponding author.

## References

[1] Harvey WT Carabelli AM Jackson B Gupta RK Thomson EC Harrison EM . SARS-CoV-2 variants, spike mutations and immune escape. Nat Rev Microbiol. (2021) 19:409–24. doi: 10.1038/s41579-021-00573-0 34075212 PMC8167834

[2] Rees-Spear C Muir L Griffith SA Heaney J Aldon Y Snitselaar JL . The effect of spike mutations on SARS-CoV-2 neutralization. Cell Rep. (2021) 34:108890. doi: 10.1016/j.celrep.2021.108890 33713594 PMC7936541

[3] Wang L Kainulainen MH Jiang N Di H Bonenfant G Mills L . Differential neutralization and inhibition of SARS-CoV-2 variants by antibodies elicited by COVID-19 mRNA vaccines. Nat Commun. (2022) 13:4350. doi: 10.1038/s41467-022-31929-6 35896523 PMC9328008

[4] Wang R Zhang Q Ge J Ren W Zhang R Lan J . Analysis of SARS-CoV-2 variant mutations reveals neutralization escape mechanisms and the ability to use ACE2 receptors from additional species. Immunity. (2021) 54:1611–1621.e5. doi: 10.1016/j.immuni.2021.06.003 34166623 PMC8185182

[5] Markov PV Ghafari M Beer M Lythgoe K Simmonds P Stilianakis NI . The evolution of SARS-cov-2. Nat Rev Microbiol. (2023) 21:361–79. doi: 10.1038/s41579-023-00878-2 37020110

[6] Dickey TH Tang WK Butler B Ouahes T Orr-Gonzalez S Salinas ND . Design of the SARS-CoV-2 RBD vaccine antigen improves neutralizing antibody response. Sci Adv. (2022) 8:eabq8276. doi: 10.1126/sciadv.abq8276 36103542 PMC9473567

[7] Li W Wang F Li Y Yan L Liu L Zhu W . Potent NTD-targeting neutralizing antibodies against SARS-CoV-2 selected from a synthetic immune system. Vaccines (Basel). (2023) 11. doi: 10.3390/vaccines11040771 37112683 PMC10143083

[8] Resende PC Naveca FG Lins RD Dezordi FZ Ferraz MVF Moreira EG . The ongoing evolution of variants of concern and interest of SARS-CoV-2 in Brazil revealed by convergent indels in the amino (N)-terminal domain of the spike protein. Virus Evol. (2021) 7. doi: 10.1093/ve/veab069 34532067 PMC8438916

[9] Greaney AJ Loes AN Crawford KHD Starr TN Malone KD Chu HY . Comprehensive mapping of mutations in the SARS-CoV-2 receptor-binding domain that affect recognition by polyclonal human plasma antibodies. Cell Host Microbe. (2021) 29:463–476.e6. doi: 10.1016/j.chom.2021.02.003 33592168 PMC7869748

[10] Li Q Zhang M Liang Z Zhang L Wu X Yang C . Antigenicity comparison of SARS-CoV-2 Omicron sublineages with other variants contained multiple mutations in RBD. MedComm (2020). (2022) 3:e130. doi: 10.1002/mco2.130 35434713 PMC8994617

[11] Kumar S Delipan R Chakraborty D Kanjo K Singh R Singh N . Mutations in S2 subunit of SARS-CoV-2 Omicron spike strongly influence its conformation, fusogenicity, and neutralization sensitivity. J Virol. (2023) 97:e0092223. doi: 10.1128/jvi.00922-23 37861334 PMC10688319

[12] Thomson EC Rosen LE Shepherd JG Spreafico R da Silva Filipe A Wojcechowskyj JA . Circulating SARS-CoV-2 spike N439K variants maintain fitness while evading antibody-mediated immunity. Cell. (2021) 184:1171–1187.e20. doi: 10.1016/j.cell.2021.01.037 33621484 PMC7843029

[13] Changrob S Fu Y Guthmiller JJ Halfmann PJ Li L Stamper CT . Cross-neutralization of emerging SARS-CoV-2 variants of concern by antibodies targeting distinct epitopes on spike. mBio. (2021) 12:e0297521. doi: 10.1128/mbio.02975-21 34781736 PMC8593667

[14] Haslwanter DA-O Dieterle MA-O Wec AZ O'Brien CM Sakharkar M Florez C . A combination of receptor-binding domain and N-terminal domain neutralizing antibodies limits the generation of SARS-CoV-2 spike neutralization-escape mutants. 12 (5) doi: 10.1128/mbio.02473-21. (2150-7511 (Electronic)). PMC8546647 34607456

[15] Cox M Peacock TP Harvey WT Hughes J Wright DW Willett BJ . SARS-CoV-2 variant evasion of monoclonal antibodies based on *in vitro* studies. Nat Rev Microbiol. (2023) 21:112–24. doi: 10.1038/s41579-022-00809-7 36307535 PMC9616429

[16] Voss WN Mallory MA Byrne PO Marchioni JM Knudson SA Powers JM . Hybrid immunity to SARS-CoV-2 arises from serological recall of IgG antibodies distinctly imprinted by infection or vaccination. 5 (8) doi: 10.1016/j.xcrm.2024.101668. (2666-3791 (Electronic)). PMC11384961 39094579

[17] Yuan M Wu NC Zhu X Lee CD So RTY Lv H . A highly conserved cryptic epitope in the receptor binding domains of SARS-CoV-2 and SARS-CoV. Sci (New York NY). (2020) 368:630–3. doi: 10.1126/science.abb7269 32245784 PMC7164391

[18] Jespersen MC Peters B Nielsen M Marcatili P . BepiPred-2.0: improving sequence-based B-cell epitope prediction using conformational epitopes. Nucleic Acids Res. (2017) 45:W24–w29. doi: 10.1093/nar/gkx346 28472356 PMC5570230

[19] Kolaskar AS Tongaonkar PC . A semi-empirical method for prediction of antigenic determinants on protein antigens. FEBS Lett. (1990) 276:172–4. doi: 10.1016/0014-5793(90)80535-q 1702393

[20] Saha S Raghava GP . Prediction of continuous B-cell epitopes in an antigen using recurrent neural network. Proteins. (2006) 65:40–8. doi: 10.1002/prot.21078 16894596

[21] Ponomarenko J Bui HH Li W Fusseder N Bourne PE Sette A . ElliPro: a new structure-based tool for the prediction of antibody epitopes. BMC Bioinf. (2008) 9:514. doi: 10.1186/1471-2105-9-514 19055730 PMC2607291

[22] Doytchinova IA Flower DR . VaxiJen: a server for prediction of protective antigens, tumour antigens and subunit vaccines. BMC Bioinf. (2007) 8:4. doi: 10.1186/1471-2105-8-4 17207271 PMC1780059

[23] Waterhouse A Bertoni M Bienert S Studer G Tauriello G Gumienny R . SWISS-MODEL: homology modelling of protein structures and complexes. Nucleic Acids Res. (2018) 46:W296–303. doi: 10.1093/nar/gky427 29788355 PMC6030848

[24] Studer G Rempfer C Waterhouse AM Gumienny R Haas J Schwede T . QMEANDisCo-distance constraints applied on model quality estimation. Bioinformatics. (2020) 36:1765–71. doi: 10.1093/bioinformatics/btaa058 31697312 PMC7075525

[25] Honorato RV Koukos PI Jiménez-García B Tsaregorodtsev A Verlato M Giachetti A . Structural biology in the clouds: The WeNMR-EOSC ecosystem. Front Mol Biosci. (2021) 8:729513. doi: 10.3389/fmolb.2021.729513 34395534 PMC8356364

[26] Honorato RV Trellet ME Jiménez-García B Schaarschmidt JJ Giulini M Reys V . The HADDOCK2.4 web server for integrative modeling of biomolecular complexes. Nat Protoc. (2024) 19:3219–41. doi: 10.1038/s41596-024-01011-0 38886530

[27] Jiménez-García B Teixeira JMC Trellet M Rodrigues J Bonvin A . PDB-tools web: A user-friendly interface for the manipulation of PDB files. Proteins. (2021) 89:330–5. doi: 10.1002/prot.26018 PMC7855443 33111403

[28] Rodrigues J Teixeira JMC Trellet M Bonvin A . pdb-tools: a swiss army knife for molecular structures. F1000Research. (2018) 7:1961. doi: 10.7490/f1000research.1117109.1 30705752 PMC6343223

[29] Pradhan A Saini S Agarwal M Kumar Y . Affinity maturation of cross-reactive CR3022 antibody against the receptor binding domain of SARS-CoV-2 via in silico site-directed mutagenesis. doi: 10.21203/rs.3.rs-92745/v1

[30] Vangone A Bonvin AMJJ . Contacts-based prediction of binding affinity in protein–protein complexes. eLife. (2015) 4:e07454. doi: 10.7554/elife.07454 26193119 PMC4523921

[31] Xue LC Rodrigues JP Kastritis PL Bonvin AM Vangone A . PRODIGY: a web server for predicting the binding affinity of protein–protein complexes. Bioinformatics. (2016) 32:3676–8. doi: 10.1093/bioinformatics/btw514 27503228

[32] Pettersen EF Goddard TD Huang CC Couch GS Greenblatt DM Meng EC . UCSF Chimera: a visualization system for exploratory research and analysis. J Comput Chem. (2004) 25:1605–12. doi: 10.1002/jcc.20084 15264254

[33] Barnes CO Jette CA Abernathy ME Dam K-MA Esswein SR Gristick HB . SARS-CoV-2 neutralizing antibody structures inform therapeutic strategies. Nature. (2020) 588:682–7. doi: 10.1038/s41586-020-2852-1 33045718 PMC8092461

[34] Lok SM . An NTD supersite of attack. Cell Host Microbe. (2021) 29:744–6. doi: 10.1016/j.chom.2021.04.010 33984277 PMC8114578

[35] Yu W Wu X Ren J Zhang X Wang Y Li C . Mechanistic insights to the binding of antibody CR3022 against RBD from SARS-CoV and HCoV-19/SARS-CoV-2: A computational study. Comb Chem High Throughput Screening. (2021) 24:1069–82. doi: 10.2174/18755402mtewuotew2 33106140

[36] Jeong HW Kim S-M Jung MK Noh JY Yoo J-S Kim E-H . Enhanced antibody responses in fully vaccinated individuals against pan-SARS-CoV-2 variants following Omicron breakthrough infection. Cell Rep Med. (2022) 3:100764. doi: 10.1016/j.xcrm.2022.100764 36182684 PMC9482837

[37] Sievers BL Chakraborty S Xue Y Gelbart T Gonzalez JC Cassidy AG . Antibodies elicited by SARS-CoV-2 infection or mRNA vaccines have reduced neutralizing activity against Beta and Omicron pseudoviruses. Sci Transl Med. (2022) 14:eabn7842. doi: 10.1126/scitranslmed.abn7842 35025672 PMC8891085

[38] Wang P Nair MS Liu L Iketani S Luo Y Guo Y . Antibody resistance of SARS-CoV-2 variants B.1.351 and B.1.1.7. Nature. (2021) 593:130–5. doi: 10.1101/2021.01.25.428137 33684923

[39] Cele S Jackson L Khoury DS Khan K Moyo-Gwete T Tegally H . Omicron extensively but incompletely escapes Pfizer BNT162b2 neutralization. 602:654–6. doi: 10.1038/s41586-021-04387-1 PMC8866126 35016196

[40] Planas D Saunders N Maes P Guivel-Benhassine F Planchais C Buchrieser J . Considerable escape of SARS-CoV-2 Omicron to antibody neutralization. Nature. (2022) 602:671–5. doi: 10.1038/s41586-021-04389-z 35016199

[41] Garcia-Beltran WF Lam EC St Denis K Nitido AD Garcia ZH Hauser BM . Multiple SARS-CoV-2 variants escape neutralization by vaccine-induced humoral immunity. Cell. (2021) 184:2372–2383.e9. doi: 10.1016/j.cell.2021.03.013 33743213 PMC7953441

[42] Chen RE Zhang X Case JB Winkler ES Liu Y VanBlargan LA . Resistance of SARS-CoV-2 variants to neutralization by monoclonal and serum-derived polyclonal antibodies. Nat Med. (2021) 27:717–26. doi: 10.1038/s41591-021-01294-w 33664494 PMC8058618

[43] Cameroni E Bowen JE Rosen LE Saliba C Zepeda SK Culap K . Broadly neutralizing antibodies overcome SARS-CoV-2 Omicron antigenic shift. Nature. (2022) 602:664–70. doi: 10.1038/s41586-021-04386-2 35016195 PMC9531318

[44] Schmidt F Muecksch F Weisblum Y Da Silva J Bednarski E Cho A . Plasma neutralization of the SARS-CoV-2 Omicron variant. N Engl J Med. (2022) 386:599–601. doi: 10.1056/nejmc2119641 35030645 PMC8757565

[45] Mader AL Tydykov L Glück V Bertok M Weidlich T Gottwald C . Omicron's binding to sotrovimab, casirivimab, imdevimab, CR3022, and sera from previously infected or vaccinated individuals. iScience. (2022) 25:104076. doi: 10.1016/j.isci.2022.104076 35309727 PMC8920075

[46] Huo J Zhao Y Ren J Zhou D Duyvesteyn HME Ginn HM . Neutralization of SARS-CoV-2 by destruction of the prefusion spike. Cell Host Microbe. (2020) 28:445–454 e6. doi: 10.2139/ssrn.3613273 32585135 PMC7303615

[47] Cerutti G Guo Y Liu L Liu L Zhang Z Luo Y . Cryo-EM structure of the SARS-CoV-2 Omicron spike. Cell Rep. (2022) 38:110428. doi: 10.1016/j.celrep.2022.110428 35172173 PMC8818377

[48] Hong Q Han W Li J Xu S Wang Y Xu C . Molecular basis of receptor binding and antibody neutralization of Omicron. Nature. (2022) 604:546–52. doi: 10.1038/s41586-022-04581-9 35228716

[49] Zhao Z Zhou J Tian M Huang M Liu S Xie Y . Omicron SARS-CoV-2 mutations stabilize spike up-RBD conformation and lead to a non-RBM-binding monoclonal antibody escape. Nat Commun. (2022) 13:4958. doi: 10.1038/s41467-022-32665-7 36002453 PMC9399999

[50] Goher SS Ali F Amin M . The delta variant mutations in the receptor binding domain of SARS-CoV-2 show enhanced electrostatic interactions with the ACE2. Med Drug Discov. (2021) 13:100114. doi: 10.1016/j.medidd.2021.100114 34901826 PMC8650763

[51] Chan KC Song Y Xu Z Shang C Zhou R . SARS-CoV-2 delta variant: Interplay between individual mutations and their allosteric synergy. (2022) 12:1742. doi: 10.3390/biom12121742 PMC9775976 36551170

[52] Baral P Bhattarai N Hossen ML Stebliankin V Gerstman BS Narasimhan G . Mutation-induced changes in the receptor-binding interface of the SARS-CoV-2 delta variant B.1.617.2 and implications for immune evasion. Biochem Biophys Res Commun. (2021) 574:14–9. doi: 10.1016/j.bbrc.2021.08.036 34425281 PMC8364676

[53] Alkhatib M Salpini R Carioti L Ambrosio Francesca A D’Anna S Duca L . Update on SARS-CoV-2 omicron variant of concern and its peculiar mutational profile. Microbiol Spectr. (2022) 10:e02732-21. doi: 10.1128/spectrum.02732-21 35352942 PMC9045195

[54] Cao Y Wang J Jian F Xiao T Song W Yisimayi A . Omicron escapes the majority of existing SARS-CoV-2 neutralizing antibodies. Nature. (2022) 602:657–63. doi: 10.1038/s41586-021-04385-3 35016194 PMC8866119

[55] Kumar S Delipan R Sharma C Jadoun J Kanjo K Singh R . Spike conformational and glycan heterogeneity associated with furin cleavage causes incomplete neutralization of SARS-CoV-2. Nat Commun. (2025) 16:10130. doi: 10.1038/s41467-025-65099-y 41257850 PMC12630640

[56] Yu W Zhong N Li X Ren J Wang Y Li C . Structure based affinity maturation and characterizing of SARS-CoV antibody CR3022 against SARS-CoV-2 by computational and experimental approaches. (2022) 14:186. doi: 10.3390/v14020186 PMC8875849 35215781

[57] Barnes CO West AP Huey-Tubman KE Hoffmann MAG Sharaf NG Hoffman PR . Structures of human antibodies bound to SARS-CoV-2 spike reveal common epitopes and recurrent features of antibodies. Cell. (2020) 182:828–842.e16. doi: 10.1016/j.cell.2020.06.025 32645326 PMC7311918

[58] Kwong PD Doyle ML Casper DJ Cicala C Leavitt SA Majeed S . HIV-1 evades antibody-mediated neutralization through conformational masking of receptor-binding sites. Nature. (2002) 420:678–82. doi: 10.1038/nature01188 12478295

[59] Casalino L Gaieb Z Goldsmith JA Hjorth CK Dommer AC Harbison AM . Beyond shielding: The roles of glycans in SARS-CoV-2 spike protein. bioRxiv. (2020). doi: 10.1101/2020.06.11.146522 PMC7523240 33140034

