# Supplementary material for: Structural and functional dissection of neutralisation differences among SARS-CoV-2 variants using antigenicity prediction and CR3022 binding analysis
Source: Front Immunol. 2026 Jul 6;17:1810369. doi: 10.3389/fimmu.2026.1810369 (PMC13381479; doi:10.3389/fimmu.2026.1810369)
Supplement: Supplementary file 1 [file DataSheet1.pdf]

1) Details of Cohort Characteristics used to study neutralization of SARS-CoV-2 Variants

To facilitate interpretation of neutralization responses across study groups, demographic characteristics, immune exposure history, timing of sample collection relative to vaccination or infection, and epidemiological context during participant enrollment are summarized below. These data are provided to clarify potential differences in antigenic exposure history, antibody waning, and circulating SARS-CoV-2 variants across cohorts.

Parameter	I group	V group	V+I group
**Sample size (n)**	25	25	25
**Age years (median IQR)**	42 (18 – 67)	38 (20 – 55)	37 (25 – 58)
**Sex (M/F)**	14/11	13/12	14/11
**Vaccination status**	Not vaccinated	Covishield (ChAdOx1 nCoV-19)	Covishield (ChAdOx1 nCoV-19)
**Number of vaccine doses**	NA	2	2
**Days between the last vaccine dose and sample collection**	NA	Mean: 40 (29-46)	NA
**Infection status**	Confirmed SARS-CoV-2 infection	No prior infection *	Breakthrough infection (post-vaccination)
**Method of infection confirmation**	RT-PCR	NA	RT-PCR
**Days between infection and sample collection**	Mean: 36 (22 – 62)	NA	Mean: 32 (30-58)
**Breakthrough infection (Yes/No)**	NA	No	Yes
**Interval: last antigenic exposure to sampling**	Infection	Vaccine	Infection
**Enrollment period (month/year range)**	May to September 2020	March to August 2021	April to July 2022

**Dominant circulating variant period, based on Indian epidemiological data**	First wave (ancestral strain predominance)	Second wave (B.1.617.2 dominant)	Third wave (BA.1 to BA.2 transition period)
**Serology screening performed**	Yes (Anti-SARS-CoV-2 spike IgG antibodies)	Yes (Anti-SARS-CoV-2 spike IgG antibodies)	Yes (Anti-SARS-CoV-2 spike IgG antibodies)
**Notes on exposure heterogeneity**	Natural infection	Ancestral spike vaccine	Hybrid immunity (vaccine + infection)

Supplementary Table S1. Demographic characteristics, immune exposure history, and enrollment chronology of study participants included in neutralization analyses. Group I: Individuals with confirmed SARS-CoV-2 infection and no prior vaccination. Group V: Individuals vaccinated with two doses of Covishield (ChAdOx1 nCoV-19) with no reported prior infection. Group V+I: Individuals with breakthrough SARS-CoV-2 infection following two doses of Covishield. Age is presented as median (min–max). Time since vaccination or infection refers to the interval between the most recent antigenic exposure and sample collection. Epidemiological periods were assigned according to dominant SARS-CoV-2 circulation patterns reported in India during the respective enrollment periods. Participants enrolled during April–July 2022 were classified within the B.1.1.529 wave period; however, exact infecting sub-lineages (BA.1 or BA.2) were not confirmed for all individuals. Anti-SARS-CoV-2 spike IgG measurement was used as a serological assessment. ***** *Prior asymptomatic SARS-CoV-2 infection cannot be ruled out due to incomplete infection history.*

2) Neutralization profile of the study groups by variants

The supplementary Figure 1 presents the individual neutralization profiles of plasma samples from the I, V, and V+I study groups against pseudoviruses expressing spike proteins of B.1, B.1.617.2, and B.1.1.529 variants. Neutralization was evaluated across serial plasma dilutions and represented as percentage neutralization versus $\log_{10}$ reciprocal dilution. These plots illustrate the heterogeneity of neutralizing antibody responses within each study group and highlight differences in neutralization breadth and potency against distinct SARS-CoV-2 variants.

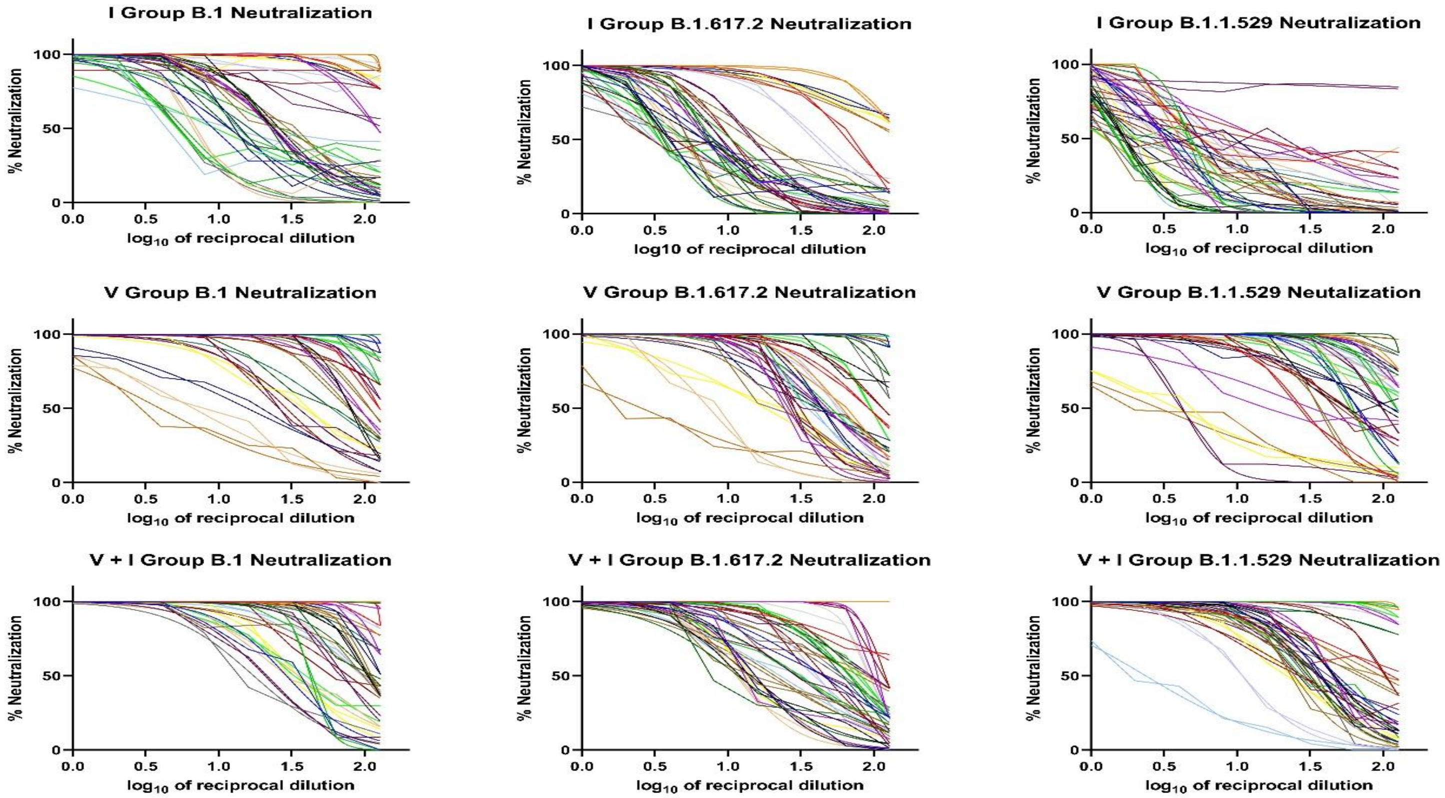

Supplementary Figure S1. Neutralization profiles of plasma samples from different study groups against SARS-CoV-2 variants: Representative neutralization curves of plasma samples from the I, V, and V+I groups against pseudoviruses bearing the spike proteins of B.1, B.1.617.2, and B.1.1.529 variants. Percentage neutralization was plotted against log_{10} reciprocal plasma dilution. Each colored line represents an individual plasma sample analysed using serial plasma dilutions in the pseudovirus neutralization assay.

3) Neutralization Curves of SARS-CoV-2 Variants by Plasma Samples with Undetectable NT_{50}

Neutralization curves with extended plasma dilutions were generated for plasma samples that initially exhibited undetectable or poorly predicted NT_{50} values within the standard assay range. Further serial dilutions enabled improved visualization of the neutralization patterns against SARS-CoV-2 variants and allowed

assessment of residual neutralizing activity at higher reciprocal plasma dilutions. These curves demonstrate the variability in neutralization potency among individual samples and support accurate interpretation of low-titer antibody responses.

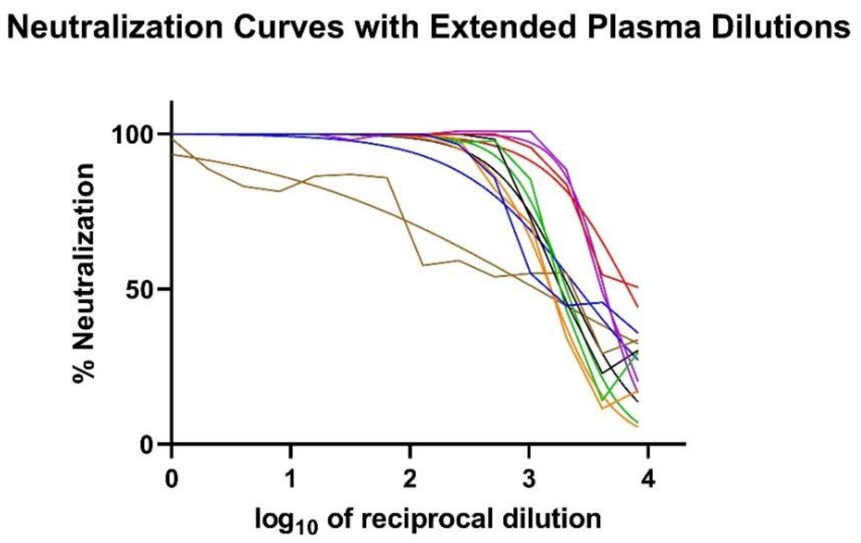

Supplementary Figure S2. Representative neutralization curves of SARS-CoV-2 variants by plasma samples with extended dilution ranges: Percentage neutralization was plotted against $\log_{10}$ reciprocal plasma dilution to evaluate neutralizing activity across an expanded dilution range. Each colored line represents an individual plasma sample.

4) Assay Reproducibility of Pseudovirus Neutralization NT_{50} Values

To assess the reproducibility of the pseudovirus neutralization assay, a subset of plasma samples was independently retested in separate experiments. The correlation analysis of $Log_{10}NT_{50}$ values obtained from the two assays demonstrated comparable neutralization measurements across experiments, supporting the consistency and reliability of the NT_{50} estimation method used in this study.

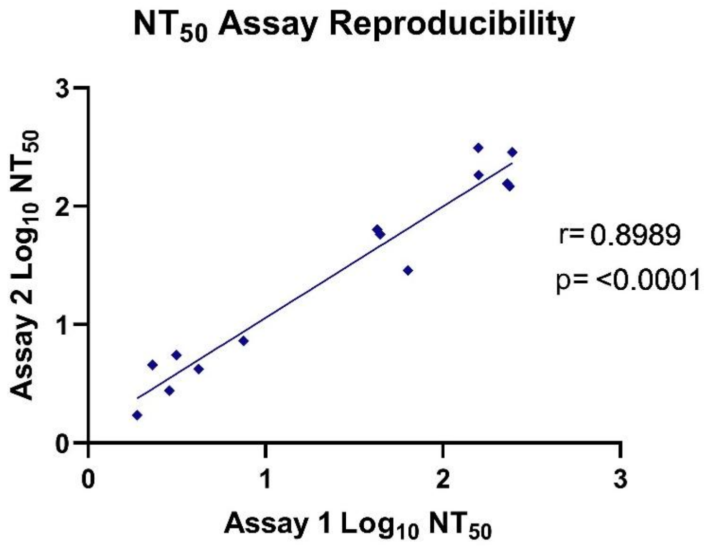

Supplementary Figure S3. Assay reproducibility analysis of NT_{50} measurements: Neutralization titers obtained from independent repeat assays of representative plasma samples showed strong agreement and correlation between experiments. Correlation analysis of $log_{10}NT_{50}$ values obtained from Assay 1 and Assay 2 was performed using Spearman's rank correlation coefficient. Each data point represents an individual plasma sample tested independently in duplicate across two assays.

5) Neutralization of SARS-CoV-2 Variants with Monoclonal Antibodies

To further examine whether the observed variant-dependent neutralization patterns were specific to CR3022 or reflected broader differences in monoclonal antibody recognition, additional neutralization analyses were performed using monoclonal antibodies MW05 (Creative Diagnostics, DMABB-JX754) and NR-55295 (BEI Resources, NIAID, NIH) against SARS-CoV-2 pseudoviruses bearing spike proteins from the Wuhan (B.1), Delta (B.1.617.2), and Omicron (B.1.1.529) variants. These experiments were included to provide comparative experimental context for the CR3022 findings and to assess the influence of spike mutations on monoclonal antibody-mediated neutralization across selected SARS-CoV-2 variants.

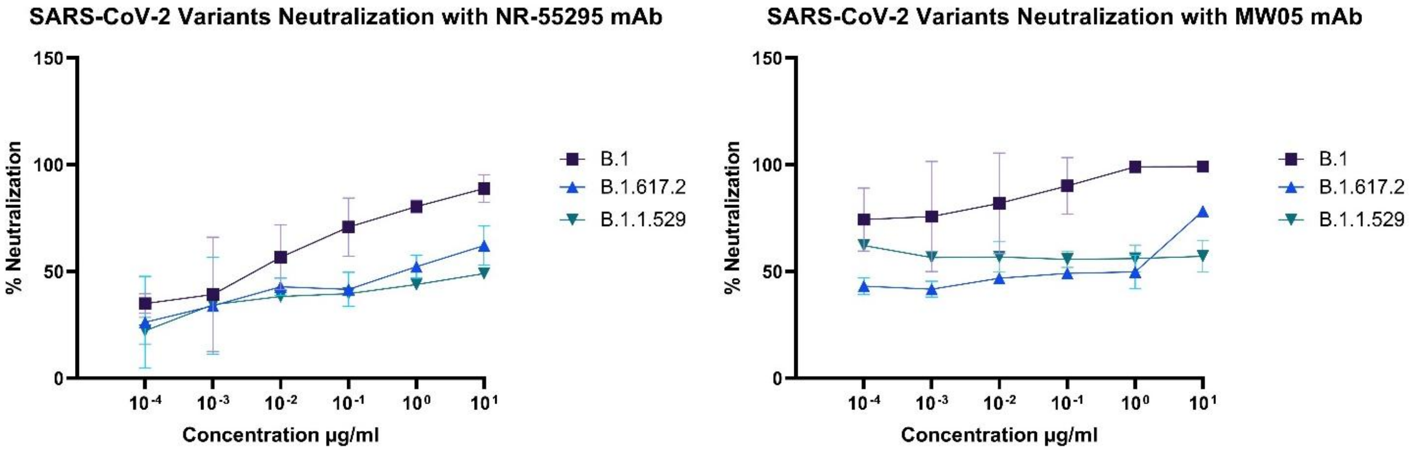

Supplementary Figure S4. Variant-dependent neutralization of B.1, B.1.617.2, and B.1.1.529 pseudoviruses by monoclonal antibodies NR-55295 and MW05

Neutralization activity of NR-55295 (left panel) and MW05 (right panel) monoclonal antibodies was evaluated against pseudoviruses bearing spike proteins from the B.1, B.1.617.2, and B.1.1.529 variants across serial antibody concentrations. Both monoclonal antibodies showed variant-dependent differences in neutralization sensitivity, with reduced neutralization observed for B.1.617.2 and B.1.1.529 variants compared to the ancestral B.1 variant. Data represent mean ± SD from two independent experiments performed in duplicate.

6) Comparative summary of predicted RBD epitopes and their distribution across SARS-CoV-2 variants

To facilitate comparison of epitope conservation and variation across SARS-CoV-2 variants, representative predicted epitopes from both the RBD and NTD are summarized in Supplementary Tables S2 and S3, respectively. Supplementary Table S3 presents RBD epitopes identified using multiple prediction algorithms, highlighting putative conserved regions observed across several variants, as well as variant-associated epitopes identified in B.1.617.2 and B.1.1.529. Some of these regions show partial overlap with previously reported antigenic sites, including the CR3022-binding region, although such correspondences should be interpreted cautiously given the in silico nature of the predictions.

	B.1 Variant RBD Linear Epitopes	Antigenicity Score	B.1.1.7 Variant RBD Linear Epitope	Antigenicity Score	B.1.351 Variant RBD Linear Epitope	Antigenicity Score	B.1.617.2 Variant RBD Linear Epitope	Antigenicity Score	B.1.1.529 Variant RBD Linear Epitope	Antigenicity Score
**ABCpred Method**	LQSYGFQPTNGVGYQP	0.5258	LQSYGFQPTYGVGYQP	0.5984	LQSYGFQPTYGVGYQP	0.5984			LRSYSFRPTYGVGHQP	0.4532
	EVRQIAPGQTGKIADY	1.3837			EVRQIAPGQTGNIADY	1.2319	EVRQIAPGQTGNIADY	1.2319	EVRQIAPGQTGNIADY	1.2319
	TGKIADYNYKLPDDFT	0.9642			PGQTGNIADYNYKLPD	1.2662	PGQTGNIADYNYKLPD	1.2662	PGQTGNIADYNYKLPD	1.2662
	FPNITNLCPFGEVFNA	0.6058							FPNITNLCPFDEVFNA	0.7579
	VQPTESIVRFPNITNL	0.4454							SVLYNLAPFFTFKCYG	0.8321
	NGVGYQPYRVVVLSFE	0.9736	YGVGYQPYRVVVLSFE	1.1621	YGVGYQPYRVVVLSFE	1.1621			YGVGHQPYRVVVLSFE	1.0055
	FSTFKCYGVSPTKLND	0.9650							FKCYGVSPTKLNDLCF	2.0332
	EGFNCYFPLQSYGFQP	0.6870			KGFNCYFPLQSYGFQP	0.7227			GFNCYFPLRSYSFRPT	1.2203
	TGCVIAWNSNNLDSKV	0.5086								
	FVIRGDEVRQIAPGQT	0.5147								
	VVLSFELLHAPATVCG	0.6184					DSKVGGNYNYRYRLFR	0.4225		
	RKSNLKPFERDISTEI	0.4298					NYNYRYRLFRKSNLKP	0.6178		
	PTKLNDLCFTNVYADS	1.2303								
**Bepipred Linear Epitope Prediction 2.0 Method**	IRGDEVRQIAPGQTG<br>KIADYNYKLPD	0.9322			IRGDEVRQIAPGQTGNIAD<br>YNYKLPD	0.8601	IRGDEVRQIAPGQTGNIADYNY<br>KLPD	0.8601	RGDEVRQIAPGQTGNIADYNYKLPD	0.9599
	NLDSKVGGNYNYLYR<br>LFRKSNLKPFERD	0.4418					LDSKVGGNYNYRYRLFRKSNLK<br>PFER	0.6395	LDSKVSGNYNYLYRLFRKSNLKPFERD<br>ISTEIYQAGNKPCNGVAGFNCYFPLRS<br>YSFRPTYGVGHQ	0.4001
	STEIYQAGSTPCNGVEGFNC<br>YFPLQSYGFQPTNGVGYQ	0.3953<br>(NON-<br>ANTIGEN)	STEIYQAGSTPCNGVEG<br>FNCYFPLQSYGFQPTYG<br>VGY	0.4038			YQAGSKPCNGVEGFNCYFPLQS<br>YGFQPTNGVGYQ	0.4834	LAPFFTFK	1.0698
									FPNITN	0.8555
**Kolaskar & Tongaonkar Antigenicity Method**	TNLCPFG	1.1812								
	TFKCYGVSPT	1.5059								
	TGCVIA	0.4716								
	CYFPLQSY	0.9394			GFNCYFPLQSY	0.9224			GFNCYFPLRS	1.5733
	YQPYRVVVLSFELLHAPATVC<br>GP	0.4697	GVGYQPYRVVVLSFELL<br>HAPATVCGP	0.7025	GVGYQPYRVVVLSFELLHA<br>PATVCGP	0.7025			GVGHQPYRVVVLSFELLHAPATVCGP	0.5988
**ElliPro: Antibody Epitope Prediction Method**	SNNLDSKVGGNYNY	0.8554					SNNLDSKVGGNYN	0.677	SNKLDSKVSGNYN	0.4955
	YGFQPTNGVGYQ	0.7136	YGFQPTYGVGYQ	0.799	YGFQPTYGVGYQ	0.799			YSFRPTYGVGHQ	0.9174
	YGVSPTKLNDLCFTN	2.0602					YGVSPTKLNDLCFT	2.2554	YGVSPTKLNDLCFT	2.2554
	RKSNLKP	1.3225								

Supplementary Table S2: B-cell epitopes predicted for RBD region of the spike protein of different SARS-CoV-2 variants (B.1, B.1.1.7, B.1.351, B.1.617.2, and B.1.1.529). Predicted linear B-cell epitope regions within the RBD of the B.1 variant and corresponding regions showing amino acid sequence differences in other variants are listed. Epitope prediction was performed using four in-silico approaches: ABCpred, a neural network-based linear epitope prediction method; BepiPred 2.0, which integrates sequence- and structure-derived features; Kolaskar and Tongaonkar antigenicity prediction based on physicochemical properties; and ElliPro, a structure-based antibody epitope prediction tool. Amino acid substitutions identified in variant-associated predicted epitope regions are highlighted in red, together with their corresponding prediction scores. Predicted epitope regions and associated prediction scores within the RBD across variants, as identified by different computational approaches, are presented in the rows of the table.

	B.1 Variant NTD Linear Epitopes	Antigenicity Score	B.1.1.7 Variant NTD Linear Epitope	Antigenicity Score	B.1.351 Variant NTD Linear Epitope	Antigenicity Score	B.1.617.2 Variant NTD Linear Epitope	Antigenicity Score	B.1.1.529 Variant NTD Linear Epitope	Antigenicity Score
**ABCpred Method**	TTRTQLPPAYTNS	0.9157	TTRTQLPPAYTNSFTRG	0.4322	TTRTQLPPAYTNSFTRGVY	0.4197	RTRTQLPPAYTN	1.1293	TTRTQLPPAYTNSFTR	0.3477
	HVSGTNGTKRFD	0.7452	SGTNGTKRFD	0.7387	AIHVSGTNGTKRFANP	0.4164	HFSGTNGTKRF	0.5101	SGTNGTKRFDN	0.5906
	LGVYYHKNNKSWMESEFRVYSSANN	0.4608	FLGVYYHKNNKSWMESEF	0.7653	FLGVYYHKNNKSWMESEFR VYSSANN	0.4143	LGVYYHKNNKSW	0.8156		
	DLEGKQGNFKNLREFVF	0.7245	MDLEGKQGNFKNLRE	1.0100	MDLEGKQGNFKNLR	1.2030	FLMDLEGKQGN	1.0187	LMDLEGKQGNFKNLR	1.1084
	SKHTPINLVRDLPQGFSAL	0.5887			VRGLPQGFSA	0.4985	INLVRDLPQGFSV	0.5054	PIIVREPEDLPQGFSA	0.5318
	RSYLTPGDSSSGWTAG	0.4016			ISYLTPGDSSSGWTA	0.6258	SYLTPGDSSSGWTA	0.5658	SYLTPGDSSSGWTA	0.5658
					ENGTIT	0.9257	EFVFKN	1.4100		
**Bepipred Linear Epitope Prediction 2.0 Method**	TTRTQLPPAYTNS	0.9157	TTRTQLPPAYTNSFTRG	0.4322	TTRTQLPPAYTNSFTRGVY	0.4197	RTRTQLPPAYTN	1.1293		
	HVSGTNGTKRFD	0.7452	SGTNGTKRFD	0.7387	AIHVSGTNGTKRFANP	0.4164	HFSGTNGTKRF	0.5101	SGTNGTKRFDN	0.5906
	LGVYYHKNNKSWMESEFRVYSSANN	0.4608	FLGVYYHKNNKSWMESEF	0.7653	FLGVYYHKNNKSWMESEFR VYSSANN	0.4143	LGVYYHKNNKSW	0.8156		
	DLEGKQGNFKNLREFVF	0.7245	MDLEGKQGNFKNLRE	1.0100	MDLEGKQGNFKNLR	1.2030	FLMDLEGKQGN	1.0187	LMDLEGKQGNFKNLR	1.1084
	SKHTPINLVRDLPQGFSAL	0.5887			VRGLPQGFSA	0.4985	EFVFKN	1.4100	PIIVREPEDLPQGFSA	0.5318
	RSYLTPGDSSSGWTAG	0.4016			ISYLTPGDSSSGWTA	0.6258	INLVRDLPQGFSV	0.5054	SYLTPGDSSSGWTA	0.5658
					ENGTIT	0.9257	SYLTPGDSSSGWTA	0.5658		
**Kolaskar & Tongaonkar Antigenicity Method**	RGVYYPDK	1.0191								
	FHAIHV	1.6766					RSSVLHST	0.5459		
	NPVLPFN	0.5863								
	QSLLIVN	0.8168								
	CNDPFLGVYYH	0.4109	CNDPFLGVY	0.4295						
	FEYVSQP	0.9073							KHTPIIVR	0.8883
	QTLLALHRSY	0.5596			TLHISYL	1.3932				
	AAYYVGYL	0.5218								
	AVDCALDP	0.7730								
**ElliPro: Antibody Epitope Prediction Method**	RSYLTPGDSSSGW	0.5173					RSYLTPGDSSSGWT	0.512	HRSYLTPGDSSSGWT	0.5842
	DLEGKQGNFK	1.3065								
	PRTFLLKYNENGTITDAVDCALDPLSET KCTLKSFTV	0.4289								
	GTTLDSKTQ	0.9425								
	IHVSGTNGTKRF	0.447	ISGTNGTKRF	0.5526			IHFSGTNGTKRF	0.7186	ISGTNGTKRF	0.5526
	CVNLTTRTQLPPAY	1.483	CVNLTTRTQLPPAYT	1.4399	CVNFTTRTQLPPAY	1.6156	CVNLRTRTQLPPAYT	1.3999	CVNLTTRTQLPPAYT	1.4399
	PINLVRD	0.5914			INLVRG	0.6539				
	VDLPIGINITR	1.4974	LPIGINI	1.6808	LPIGINI	1.6808	LPIGINI	1.6808	VDLPIGINI	1.3808

Supplementary Table S3. B Cell Epitope predicted for the NTD region of the Spike protein of different variants (B.1, B.1.1.7, B.1.351, B.1.617.2, and B.1.1.529). Predicted linear B-cell epitope regions within the NTD of the B.1 variant and corresponding regions showing amino acid sequence differences in other variants are listed. Epitope prediction was performed using four in-silico approaches: ABCpred, a neural network-based linear epitope prediction method; BepiPred 2.0, which integrates sequence- and structure-derived features; Kolaskar and Tongaonkar antigenicity prediction based on physicochemical properties; and ElliPro, a structure-based antibody epitope prediction tool. Amino acid substitutions identified in variant-associated predicted epitope

regions are highlighted in red together with their corresponding prediction scores. Predicted epitope regions and associated prediction scores within the NTD across variants, as identified by different computational approaches, are presented in the rows of the table.

7) Structural comparison of representative predicted variant-specific B-cell epitope regions within the RBD and NTD of SARS-CoV-2

To further visualize predicted epitope differences among variants, representative B-cell epitope regions identified in B.1.617.2 and B.1.1.529 were mapped onto RBD and NTD structures. The structural representations provide contextual comparison of predicted conserved and variant-associated epitopes relative to previously reported antigenic regions and are intended as exploratory visualization rather than experimental validation of antibody binding.

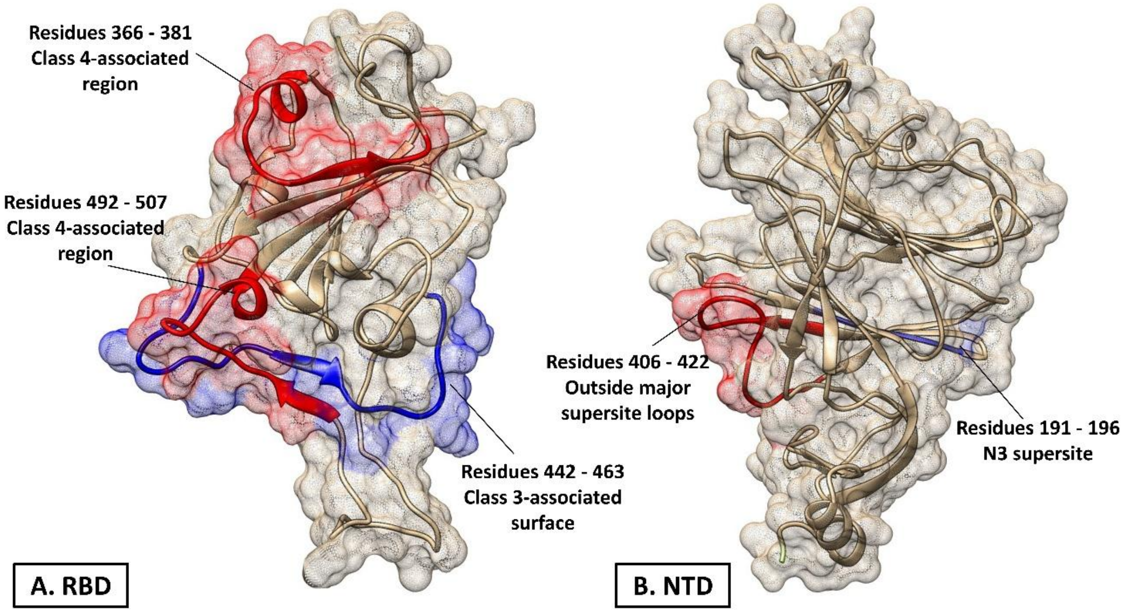

Supplementary Figure S5. Structural comparison of representative predicted B-cell epitope regions between B.1.617.2 and B.1.1.529 variants within the RBD and NTD of SARS-CoV-2. Ribbon and surface representations of the spike RBD and NTD are shown with representative predicted epitope regions highlighted for comparative visualization. (A) RBD showing representative variant-associated predicted epitope regions identified in B.1.617.2 (Blue) and B.1.1.529 (Red), mapped for comparison with previously described antibody class-associated surfaces. Although several predicted epitopes localized to broadly similar structural regions, differences in amino acid composition were observed between variants, including regions corresponding to the reported Class 4 (CR3022-associated) and Class 3-associated surfaces. (B) NTD showing representative variant-associated predicted epitope regions identified in B.1.617.2 (Blue), and B.1.1.529 (Red),

mapped relative to previously described NTD antigenic supersite regions and adjacent surface-exposed regions. Predicted epitopes occupied overlapping or neighbouring structural locations but exhibited sequence differences between variants, illustrating redistribution of the predicted epitope landscape across the NTD. Residue numbering corresponds to the Wuhan-Hu-1 reference spike sequence (GenBank accession NC_045512). Structural visualization was performed using UCSF Chimera. Antibody class and supersite annotations are approximate and provided for contextual interpretation only; highlighted regions represent computationally predicted epitopes.

8) Modelling of the RBD of the spike glycoprotein of SARS-CoV-2 variants

The QMEANDisCo Global score and Global Model Quality Estimation (GMQE) values were used to assess the predicted structural models. The structural validation metrics are summarized in Supplementary Table S4. The GMQE scores ranged from 0.73 to 0.74, suggesting generally reliable model quality. The QMEANDisCo Global scores indicated that the overall structural features were consistent with those expected for protein models of comparable size and complexity, although these metrics should be interpreted as predictive rather than definitive indicators of native-like structure. Ramachandran plot analysis using PROCHECK showed acceptable stereochemical properties, with more than 85% of residues located in the most favoured regions, supporting reasonable backbone geometry within the modelled structures.

Model Name	Sequence Identity	GMQE	QMEANDisCo Global	Ramchandran Plot: Residues in Most Favourable Region	Ramchandran Plot: Residues in Additional Allowed Region
**B.1 RBD**	100.00	0.74	0.77± 0.06	86.3%	13.1%
**B.1.1.7 RBD**	99.49	0.74	0.77± 0.06	86.3%	13.1%
**B.1.351 RBD**	98.46	0.74	0.77± 0.06	86.3%	13.1%
**B.1.617.2 RBD**	98.46	0.73	0.76± 0.06	85.7%	14.3%
**B.1.1.529 RBD**	92.31	0.73	0.76± 0.06	85.3%	14.1%

Supplementary Table S4. Structural validation metrics for the homology modelled RBD region of SARS-CoV-2 variants generated using SWISS-MODEL. Sequence identity, GMQE, and QMEANDisCo global scores were obtained from the SWISS-MODEL output and used as general indicators of model reliability. Ramachandran plot analysis performed using PROCHECK was used to assess backbone dihedral angle distributions. The stereochemical quality of the models was evaluated based on the proportion of residues in the most favoured and additionally allowed regions. No residues were observed in strongly disallowed regions in any of the models, suggesting overall acceptable stereochemical geometry, although these validation metrics should be interpreted within the limitations of comparative modelling.

9) Computational Docking of CR3022 with RBDs of SARS-CoV-2 Variants

To explore potential structural features associated with CR3022 interactions across SARS-CoV-2 lineages, HADDOCK 2.4 docking was performed using the RBDs of B.1, B.1.1.7, B.1.351, B.1.617.2, and B.1.1.529 variants. The docking analysis showed differences in modelled interaction parameters among variants (Table 2). More favourable HADDOCK scores were observed for the B.1 (–151.4 ± 4.3) and B.1.1.529 (–151.0 ± 1.4) complexes, whereas B.1.351 (–143.9 ± 1.5) and B.1.1.7 (–144.4 ± 6.6) showed comparatively less favourable scores. RMSD values were low across clusters (0.4–0.7 Å), indicating similar docking conformations within the modelled complexes.

Differences were also observed in individual energy terms and interface descriptors. The B.1 model showed more favourable electrostatic energy values (–498.6 ± 11.0), while B.1.617.2 displayed comparatively lower van der Waals energy values (–83.9 ± 2.1) and a larger buried surface area (2546.9 ± 21.9). B.1.1.7 and B.1.351 exhibited comparatively less favourable electrostatic contributions relative to other modelled complexes. Although B.1.1.529 showed an overall HADDOCK score similar to B.1, differences in individual energy components were observed.

Overall, these computational docking results suggest that amino acid differences among RBD variants may influence the modelled CR3022-RBD interaction interface. However, these observations represent in-silico predictions and should not be interpreted as direct measurements of binding affinity or antibody recognition.

	**B.1 RBD**	**B.1.1.7 RBD**	**B.1.351 RBD**	**B.1.617.2 RBD**	**B.1.1.529 RBD**
**HADDOCK score**	-151.4 +/- 4.3	-144.4 +/- 6.6	-143.9 +/- 1.5	-147.1 +/- 5.9	-151.0 +/- 1.4
**Cluster size**	113	113	125	108	114
**RMSD from the overall lowest-energy structure**	0.6 +/- 0.3	0.7 +/- 0.4	0.6 +/- 0.3	0.4 +/- 0.3	0.6 +/- 0.4
**Van der Waals energy**	-74.0 +/- 5.4	-79.2 +/- 4.6	-79.1 +/- 5.1	-83.9 +/- 2.1	-79.5 +/- 9.2
**Electrostatic energy**	-498.6 +/- 11.0	-458.0 +/- 44.3	-468.2 +/- 6.7	-481.1 +/- 6.3	-463.3 +/- 21.0
**Desolvation energy**	-13.4 +/- 3.7	-14.4 +/- 3.5	-11.2 +/- 3.1	-12.0 +/- 2.7	-13.1 +/- 2.5
**Restraints violation energy**	356.5 +/- 22.3	407.9 +/- 59.5	400.6 +/- 30.3	450.0 +/- 58.1	342.8 +/- 106.4

**Buried Surface Area**	2437.6 +/- 77.1	2420.7 +/- 167.3	2488.6 +/- 72.3	2546.9 +/- 21.9	2402.0 +/- 114.4
**Z-Score**	-2.1	-2.1	-2.5	-2.2	-2.1

Supplementary Table S5. Predicted docking parameters for CR3022 antibody interactions with the RBDs of major SARS-CoV-2 variants generated using HADDOCK. This table summarizes computational docking outputs for CR3022 interactions with the receptor-binding domain (RBD) of SARS-CoV-2 variants B.1, B.1.1.7, B.1.351, B.1.617.2, and B.1.1.529. Reported parameters include HADDOCK score, cluster size, RMSD relative to the lowest-energy model within the cluster, van der Waals energy, electrostatic energy, desolvation energy, restraints violation energy, and buried surface area (BSA). Lower HADDOCK scores and more favourable interaction energy terms may suggest greater predicted compatibility of the modelled antibody–RBD interaction under the docking conditions used. Observed differences across variants should be interpreted as computational estimates that may reflect the influence of amino acid substitutions on modelled interface properties and are not direct measures of binding affinity or experimental interaction strength.
